# Shoot chloride exclusion and salt tolerance in grapevine is associated with differential ion transporter expression in roots

**DOI:** 10.1186/s12870-014-0273-8

**Published:** 2014-10-25

**Authors:** Sam W Henderson, Ute Baumann, Deidre H Blackmore, Amanda R Walker, Rob R Walker, Matthew Gilliham

**Affiliations:** Australian Research Council Centre of Excellence in Plant Energy Biology, School of Agriculture, Food and Wine, & Waite Research Institute, University of Adelaide, PMB1, Glen Osmond, South Australia, 5064 Australia; Australian Centre for Plant Functional Genomics, South Australia, 5064 Australia; CSIRO Plant Industry, PO Box 350, Glen Osmond, South Australia 5064 Australia

**Keywords:** ABA signalling, ACA, CAX, mRNA, Salt overly sensitive (SOS), Woody perennial

## Abstract

**Background:**

Salt tolerance in grapevine is associated with chloride (Cl^−^) exclusion from shoots; the rate-limiting step being the passage of Cl^−^ between the root symplast and xylem apoplast. Despite an understanding of the physiological mechanism of Cl^−^ exclusion in grapevine, the molecular identity of membrane proteins that control this process have remained elusive. To elucidate candidate genes likely to control Cl^−^ exclusion, we compared the root transcriptomes of three *Vitis* spp. with contrasting shoot Cl^−^ exclusion capacities using a custom microarray.

**Results:**

When challenged with 50 mM Cl^−^, transcriptional changes of genotypes 140 Ruggeri (shoot Cl^−^ excluding rootstock), K51-40 (shoot Cl^−^ including rootstock) and Cabernet Sauvignon (intermediate shoot Cl^−^ excluder) differed. The magnitude of salt-induced transcriptional changes in roots correlated with the amount of Cl^−^ accumulated in shoots. Abiotic-stress responsive transcripts (e.g. heat shock proteins) were induced in 140 Ruggeri, respiratory transcripts were repressed in Cabernet Sauvignon, and the expression of hypersensitive response and ROS scavenging transcripts was altered in K51-40. Despite these differences, no obvious Cl^−^ transporters were identified. However, under control conditions where differences in shoot Cl^−^ exclusion between rootstocks were still significant, genes encoding putative ion channels *SLAH3*, *ALMT1* and putative kinases *SnRK2.6* and *CPK*s were differentially expressed between rootstocks, as were members of the *NRT1* (*NAXT1 and NRT1.4*), and *CLC* families.

**Conclusions:**

These results suggest that transcriptional events contributing to the Cl^−^ exclusion mechanism in grapevine are not stress-inducible, but constitutively different between contrasting varieties. We have identified individual genes from large families known to have members with roles in anion transport in other plants, as likely candidates for controlling anion homeostasis and Cl^−^ exclusion in *Vitis* species. We propose these genes as priority candidates for functional characterisation to determine their role in chloride transport in grapevine and other plants.

**Electronic supplementary material:**

The online version of this article (doi:10.1186/s12870-014-0273-8) contains supplementary material, which is available to authorized users.

## Background

Grapevine (*Vitis vinifera* L.), used for wine, table grape and dried grape production, is an economically important crop plant that is moderately sensitive to salinity [1]. Grapevine salt stress symptoms include reduced stomatal conductance, reduced photosynthesis [2,3] and leaf burn [4], which are generally associated with increases in shoot chloride (Cl^−^) rather than sodium (Na^+^) concentrations [3]. Reduced vigour [5] and reduced yield [6] are further effects of salt stress, with a strong positive correlation between the two [5]. Certain non-*vinifera Vitis* spp. rootstocks are used commercially to constrain shoot Cl^−^ accumulation and confer improved salt tolerance to grafted *V. vinifera* scions [7,8]. Despite a detailed understanding of the physiology of shoot Cl^−^ accumulation in grapevine and other plants, the genes responsible for this process across the plant kingdom are not known [9]. This is in contrast to the control of long-distance Na^+^ transport in plants where numerous reports have targeted known genes in order to improve the salt tolerance of plants, particularly cereals e.g. [10-13]. Due to extensive natural variation in the shoot Cl^−^ exclusion capacity of *Vitis* spp. [14,15] grapevine represents an ideal model to identify candidate genes involved in controlling shoot Cl^−^ exclusion.

Solutes travel from the roots to the shoot in the xylem. Physiological studies using radiotracers and fluorescent dyes in grapevine have indicated that the transfer of solutes to the xylem apoplast involves a symplastic step, and that rootstocks confer Cl^−^ exclusion to a grafted scion by reducing net xylem loading of Cl^−^ [15,16]. Patch clamp studies of xylem parenchyma protoplasts identified the passive quickly activating anion conductance (X-QUAC) as capable of catalysing the majority of Cl^−^ flux to the xylem of barley roots [17]. Cl^−^ entry to the root xylem is down-regulated by abscisic acid (ABA), as demonstrated by ^36^Cl^−^ fluxes in excised roots and whole seedlings of barley [18], and reduces X-QUAC of maize xylem parenchyma cells [19]. Given that ABA rises in concentration in plant roots exposed to salt stress [20], anion transporters expressed in cells that surround the root xylem, especially those that change activity when plants are salt treated are likely to be good targets to explore for improving our understanding how shoot Cl^−^ exclusion is conferred.

There have been a limited number of studies that have provided insights to the genetic elements that control long-distance transport of Cl^−^. Like grapevine, *Citrus* spp. are moderately salt-sensitive woody perennial crops frequently grown on salt-excluding rootstocks. Brumos *et al.* [21] compared the partial leaf transcriptomes of *Citrus* rootstocks Cleopatra mandarin (a good shoot Cl^−^ excluder) and Carrizo citrange (a poor shoot Cl^−^ excluder) exposed to NaCl and KCl stress using a cDNA microarray covering 6,875 putative unigenes. They concluded that a nitrate (NO_3_^−^) transporter with homology to *GmNRT1-2* from soybean was differentially expressed between rootstocks and therefore was deemed a candidate gene for influencing Cl^−^ movement. Using the same germplasm, Brumos *et al.* [22] used quantitative PCR to measure root expression of three candidate genes for the control of long-distance Cl^−^ transport derived from the literature. Candidates included a homolog of a cation chloride co-transporter (*CcCCC1*), *CcICln1* (a putative regulator of chloride channel conductance) and *CcSLAH1*, a homolog of the plant guard cell slow anion channels (SLAC) [22]. Of these genes *SLAH1* was more highly expressed in the chloride accumulating rootstock under 90 mM NaCl stress. In guard cells, SLAC chloride channels meditate ABA induced passive Cl^−^ efflux causing stomatal closure [23,24]. SLAC homologs (SLAH) in plant roots are therefore particularly interesting candidates for xylem loading of Cl^−^, but their role in roots remains uncharacterised. *CCC* was proposed to regulate retrieval of Na^+^, K^+^ and Cl^−^ from the Arabidopsis root xylem but was not regulated transcriptionally by salt [22,25]. Furthermore, questions remain as to how CCC can act directly in xylem loading on the plasma membrane due to unfavourable electrochemical gradients [9]. *ICln1* homologs from rat and *Xenopus laevis* elicit Cl^−^ currents in voltage clamp experiments [26]. In *Citrus*, *ICln1* exhibited strong repression in the Cl^−^ excluder after application of 4.5 mM Cl^−^ [22]. However, *ICln* proteins from plants remain uncharacterised. Whilst these genes are good candidates for regulating Cl^−^ transport in *Citrus*, analyses of entire root transcriptomes is likely to provide a more complete list of factors that mediate long-distance transport of Cl^−^.

Gene expression studies of *V. vinifera* have been greatly aided by the draft genome sequence of Pinot Noir inbred line PN40024 [27,28]. These studies have concentrated on berry development [29,30], leaf responses to heat stress [31] and to UV radiation [32]. The most comprehensive grapevine expression study to date compared the transcriptome of 54 samples representing different vegetative and reproductive organs at various developmental stages [33]. Although abiotic stress was not analysed in this study, grapevine roots were found to express more organ-specific transcripts than leaves [33]. This is consistent with findings from Tillett *et al.,* [34] who compared large-scale EST libraries from roots and shoots of Cabernet Sauvignon and identified 135 root enriched transcripts. These findings indicate that shoot expression analyses of grapevine, while useful, might not give a complete picture of root gene expression patterns, and therefore studies into root responses to abiotic stresses are required. Two microarray studies have examined the effect of salinity stress on transcript levels of Cabernet Sauvignon shoot tips [35,36]. Increased levels of a transcript encoding a putative *NRT* were observed, while decreased expression of a chloride channel (*CLC*) with sequence similarity to Arabidopsis *AtCLC-d* was detected by two probe sets, but this was not statistically significant [36].

We performed a comparative microarray of mRNAs derived from roots of salt stressed and control Cabernet Sauvignon, 140 Ruggeri and K51-40 rooted leaves as an unbiased method to identify candidates for long-distance transport of Cl^−^. We aimed to test the hypothesis that the differences in Cl^−^ exclusion between rootstocks 140 Ruggeri and K51-40 could be due to expression differences in genes that encode membrane transport proteins which facilitate root-to-shoot Cl^−^ translocation. The identification of genes that prevent excessive shoot Cl^−^ accumulation in grapevine will facilitate continued rootstock development by providing genetic markers for rootstock breeding programs. Furthermore, this study will aid a greater understanding of plant Cl^−^ homeostasis by using grapevine as a model species to elucidate genes that underpin the Cl^−^ exclusion trait in plants in general.

## Methods

### Preparation of rooted-leaves

Grapevine, being a woody perennial crop, is challenging to use in controlled conditions experiments, especially where large amounts of material and multiple replicates are required. We therefore used the method of Schachtman and Thomas [37] where leaves are excised from a parent plant and grown as rooted-leaves. This is consistent with previous studies of Cl^−^ accumulation in vines, where it was demonstrated that root and leaf phenotypes acquired with this system are similar to field observations [15,16]. Rooted leaves were established from pot-grown grapevines of K51-40 (*Vitis champinii* X *Vitis riparia*), 140 Ruggeri (*Vitis berlandieri* X *Vitis rupestris*) and Cabernet Sauvignon (*Vits vinifera*) established from cuttings and maintained in a glasshouse as described previously [15]. After approximately 3 weeks, rooted-leaves were transferred to aerated hydroponic tanks containing modified Hoagland Solution with the following nutrients (in mM) for a two-week pre-treatment period: KNO_3_, 1.0; Ca(NO_3_)_2_ · 4H_2_O, 1.0; MgSO_4_ · 7H_2_O, 0.4; KH_2_PO_4_, 0.2; H_3_BO_3_, 4.6 × 10^−2^; MnCl_2_ · 4H_2_O, 9.1 × 10^−3^; ZnSO_4_ · 7H_2_O, 7.6 × 10^−4^; CuSO_4_ · 5H_2_O, 3.2 × 10^−4^; Na_2_MoO_4_ · 2H_2_O, 2.4 × 10^−4^; EDTA-Fe-Na, 7.1 × 10^−2^ (pH 6.5) [15].

### Response of intact rooted-leaves to short term salinity

Rooted-leaves of K51-40, 140 Ruggeri and Cabernet Sauvignon were subjected to nutrient solution only (control) or to 50 mM Cl^−^ (Na^+^: Ca^2+^: Mg^2+^ = 6:1:1) in nutrient solution for 4 days. At harvest, the rooted-leaves of each genotype were washed in de-ionised water, blotted dry with paper towel, weighed, then separated into lamina, petiole and roots. Fresh weights of all plant parts were also obtained. Samples were divided equally for RNA extraction and ion composition analysis. Samples for RNA extraction were snap frozen in liquid nitrogen and stored at minus 80°C. Root, petiole and lamina samples for ion analysis were weighed before being dried in an oven at 60°C and retained for Cl^−^ analysis.

For stele and cortex expression studies roots were salt-treated and harvested as described above, lateral roots were removed from main roots and then cortex was stripped from stele of the main root using fine tweezers. Three biological replicates were harvested, each consisting of dissected tissue from three rooted-leaves. Tissue samples were immediately frozen in liquid nitrogen and stored at minus 80°C for RNA extraction.

### Ion analyses

Laminae, petiole and root samples were dried at 60°C for at least 72 h and ground to a fine powder using a mortar and pestle. Cl^−^ concentration was measured by silver ion titration with a chloridometer (Model 442–5150, Buchler Instruments, Lenexa, Kansas, USA) from extracts prepared by digesting 20–100 mg dry samples in 4 mL of acid solution containing 10% (v/v) acetic acid and 0.1 M nitric acid overnight before analysis.

### RNA extraction

Frozen root tissues were ground to a fine powder in liquid nitrogen using a mortar and pestle. RNA was extracted using the Spectrum Plant Total RNA Kit (Sigma, St. Louis, Missouri, USA) following the manufacturer’s protocol. RNA was DNase I treated with Turbo DNA-free (Life Technologies, Carlsbad, California, USA) for 1 hour at 37°C to remove contaminating genomic DNA. RNA was precipitated at minus 80°C overnight in 5 volumes of 100% ethanol (v/v) and 1/10 volumes of 3 M NaOAC. After ethanol precipitation, RNA was resuspended in nuclease free water and analysed on a NanoDrop 1000 spectrophotometer (Thermo Fisher Scientific, Waltham, Massachusetts, USA). Only RNA samples with 260/280 and 260/230 absorbance ratios greater than 1.8 were used. RNA integrity was screened on a Bioanalyzer 2100 (Agilent Technologies, Santa Clara, California, USA) and only RNA samples with an RNA integrity number (RIN) above 8.5 were used.

### Microarray chip design, labelling and hybridisation

Custom 8x60K gene expression microarrays were designed using eArray (Release 7.6) (Agilent Technologies). Oligonucleotide probes (60-mers) were designed against 26,346 annotated *V. vinifera* transcripts from the 12x Genoscope build available from http://www.genoscope.cns.fr/externe/GenomeBrowser/Vitis/. The Agilent 60-mer probe format is considered more tolerant to sequence mismatches than 25-mers, and more suitable for analysis of polymorphic DNA sequences [38]. Also, the use of a custom Agilent expression array enabled us to print a subset of probes for 90 putative anion transporters multiple times on the array (Additional file 1). This multi-probe approach increases the robustness of the expression values obtained when the probes for these genes are averaged. Probes that detect differential gene expression many times show a greater probability of genuine differential expression when the B-statistic probability (log-odds) of differential gene expression is calculated. The higher the B-statistic, the greater the chance that the gene is differentially expressed (B-statistic = 0 represents 50:50 chance of differential gene expression).

Twenty-two microarrays were used which consisted of 4 biological replicates for Cabernet Sauvignon (±50 mM Cl^−^), 4 biological replicates of K51-40 (±50 mM Cl^−^) and 3 biological replicates of 140 Ruggeri (±50 mM Cl^−^). Each biological replicate consisted of roots from 4 rooted-leaves pooled together. Single colour labelling, hybridisations and image analysis were performed at the Ramaciotti Centre for Gene Function Analysis (University of New South Wales, Australia).

### Functional annotation of genes

Gene functional annotation, which included InterPro descriptions, Gene Ontology terms and Arabidopsis orthologs, was obtained from BioMart at EnsemblPlants (plants.ensembl.org/biomart/martview/)*.* Additional functional annotation was gathered from Grimplet *et al.* [39], and this annotation was used for the tables and figures presented in this manuscript.

### Microarray data analysis

Scanned images were analysed with Feature Extraction Software 10.7.3 (Agilent Technologies, Santa Clara, California, USA) and the Cy3 median signal intensities for each spot on the arrays were imported into R for further processing. The data was log(2) transformed and quantile normalized. Since the microarray hybridizations were performed at different dates we observed batch effects that we corrected for with the ComBat package [40]. The quality of the microarray hybridisation and reproducibility amongst biological replicates was validated using arrayQualityMetrics version 3.12.0 [41]. Differentially expressed genes were identified using the Linear Model for Microarray Data (LIMMA) package [42], and the Benjamini and Hochberg correction method was applied to account for multiple testing [43]. To filter the probes, the probe sequences were blasted against the predicted cDNAs of the 12xV1 genome sequence at EnsemblPlants. Probes with an e-value ≥1×10^−10^ and probes that showed no blast hit were excluded from the initial analyses. Gene expression changes were considered significant when a threshold fold change of greater than or equal to 1.41 was reached (log(2) FC ±0.5) and a false discovery rate (FDR) corrected probability of *P* ≤0.05. The raw data for the microarray are available at the Gene Expression Omnibus database (http://www.ncbi.nlm.nih.gov/geo/) under accession number GSE57770.

Hierarchical clustering and co-expression analysis was performed using Genesis 1.7.6 [44] using tab delimited text files of the log(2) fold change values of gene expression of averaged probes. Transcripts and experiments were clustered using the average linkage method. Singular enrichment analysis was performed using Agrigo [45]. At the time of writing, the Agrigo server is incompatible with 12xV1 *V. vinifera* gene IDs. Therefore transcripts that were differentially expressed (identified after filtering) were entered into the Agrigo server using the 12xV0 transcript ID’s (Genoscope). The hypergeometric method with Hochberg (FDR) multi-test adjustment was used to identify statistically significant GO terms (*P* <0.05).

### Phylogenetic analyses

*V. vinifera* protein sequences of interest were obtained from EnsemblPlants using the 12xV1 gene IDs. *V. vinifera* amino acid sequences were used as a query in a protein-protein BLAST (blastp) at the National Centre for Biotechnology Information (NCBI) against non-redundant protein sequences limited to *Arabidopsis thaliana* (taxid: 3702). Arabidopsis sequences with the best total score were reciprocally blasted at EnsemblPlants against the *Vitis vinifera* peptide database. Arabidopsis and grapevine sequences that were obtained using this approach were aligned using Clustal W2 [46]. Phylogenetic trees were generated with Geneious 6.1.2 (Biomatters) using the neighbour-joining method and the Jukes-Cantor genetic distance model. A consensus tree was generated by re-sampling 1000 times using the bootstrap method. Branch lengths are proportional to the amount of divergence between nodes in units of substitutions per site. Gene identifiers for the protein sequences used are shown in Additional file 2, while the multiple sequence alignment is shown in Additional file 3.

### Quantitative real-time PCR (qRT-PCR)

One microgram of total RNA was reverse transcribed in a 20 μL reaction using iScript cDNA Synthesis Kit (Bio-Rad, Hercules, California, USA). The procedure was modified from the manufacturer’s to include an initial RNA denaturation step of 65°C for 5 minutes then incubation on ice for 1 minute, and cDNA synthesis step of 42°C for 1 hour. cDNA was diluted 1 in 5. Two microliters of cDNA was used as a template for PCR and qRT-PCR reactions. PCR targets were first amplified from cDNA using KAPA taq (KAPA Biosystems, Woburn, Massachusetts, USA) following manufacturer’s procedures. Fragments of the correct size and target were confirmed by agarose gel and sequencing. PCR fragments, or linearised plasmid containing the PCR fragment, were serially diluted and used as a template for qRT-PCR in duplicate. Standard curves were generated using iCycler iQ optical system software version 3.1 (Bio-Rad), which also calculates the reaction efficiency of each primer pair using the formula E = 10^-1/slope^. qRT-PCR was performed on a Bio-Rad iCycler. Reactions consisted of 250 nM forward and reverse primer, 1x KAPA SYBR FAST qPCR Master Mix (KAPA Biosystems), and 2 μL of diluted cDNA. Reactions were performed in triplicate following a three-step protocol consisting of 40 cycles of the following: 95°C 15 sec, 56°C 20 sec, 72°C 10 sec (plus data acquisition). Melt curve analysis was performed by heating PCR products from 52°C to 92°C for 20 seconds increasing at 0.5°C per cycle with continuous fluorescence detection. Relative expression ratios were calculated using the primer pair efficiency and the formula described by Pfaffl [47], with the geometric mean of *VvActin1*, *VvUbiquitin-L40* and *VvElongation-factor-1-α* used as the reference for normalisation [48]. Normalised expression values were transformed to log(2) values for comparison with microarray data. Primers used for qRT-PCR are listed in Additional file 4. Primers were designed using Primer3 [49]. Primers were designed to amplify single products from the target gene between 140 and 250 bp with an optimal GC content of 50% and, where possible, designed to span an intron to ensure that cDNA targets were amplified. Before their use, primers were screened for potential non-selective amplification using PrimerBLAST at NCBI against the Refseq RNA database limited to *Vitis* species.

## Results

### Salt treatment, grapevine growth and ion accumulation

**Figure 1:**
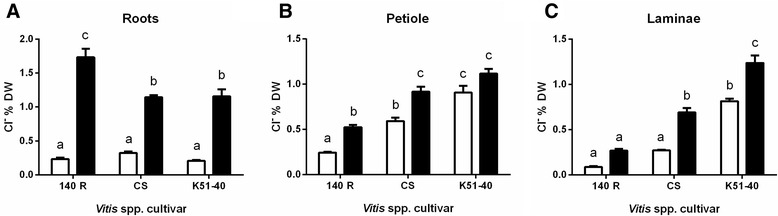
**Differential chloride accumulation in tissues of different** ***Vitis*** **spp.** Chloride concentration (% dry weight) in the roots **(A)**, petiole **(B)** and laminae **(C)** of hydroponically grown rooted-leaves under control (white bars) or 50 mM Cl^−^ (black bars) conditions. Bars represent the mean ± SEM of 4 biological replicates. Statistical significance was determined by one-way ANOVA with Bonferroni post-hoc test (*P* <0.05). CS = Cabernet Sauvignon, 140 R = 140 Ruggeri.

Following 4-days of 50 mM Cl^−^ treatment, roots of 140 Ruggeri retained significantly more Cl^−^ compared to those of Cabernet Sauvignon and K51-40 (Figure 1A). Conversely, Cabernet Sauvignon and K51-40 petioles and laminae accumulated significantly more Cl^−^ compared to 140 Ruggeri (Figure 1B and C). K51-40 accumulated the highest amount of Cl^−^ in aerial tissues under salt stress (Figure 1B and C). Under control conditions, 140 Ruggeri also accumulated significantly less petiole and laminae Cl^−^ than K51-40, indicating that the Cl^−^ exclusion mechanism may be active in low Cl^−^ conditions (Figure 1B and C). Overall, the shoot Cl^−^ accumulation of varieties can be expressed as 140 Ruggeri < Cabernet Sauvignon < K51-40.

### Validation of microarray data using real-time quantitative PCR (qRT-PCR)

To validate the microarray expression data and further quantify mRNA expression levels, we measured the expression of 12 genes by qRT-PCR and compared the datasets. Expression ratios of genes from control and 50 mM Cl^−^ treated samples were analysed by linear regression and an R^2^ value of 0.88 was observed, indicating good correlation (Additional file 5a). Similarly, qRT-PCR and microarray ratios for 12 genes were compared between varieties under control conditions, which provided an R^2^ value of 0.89, also demonstrating good correlation (Additional file 5b).

### Differentially expressed genes due to chloride stress

**Figure 2:**
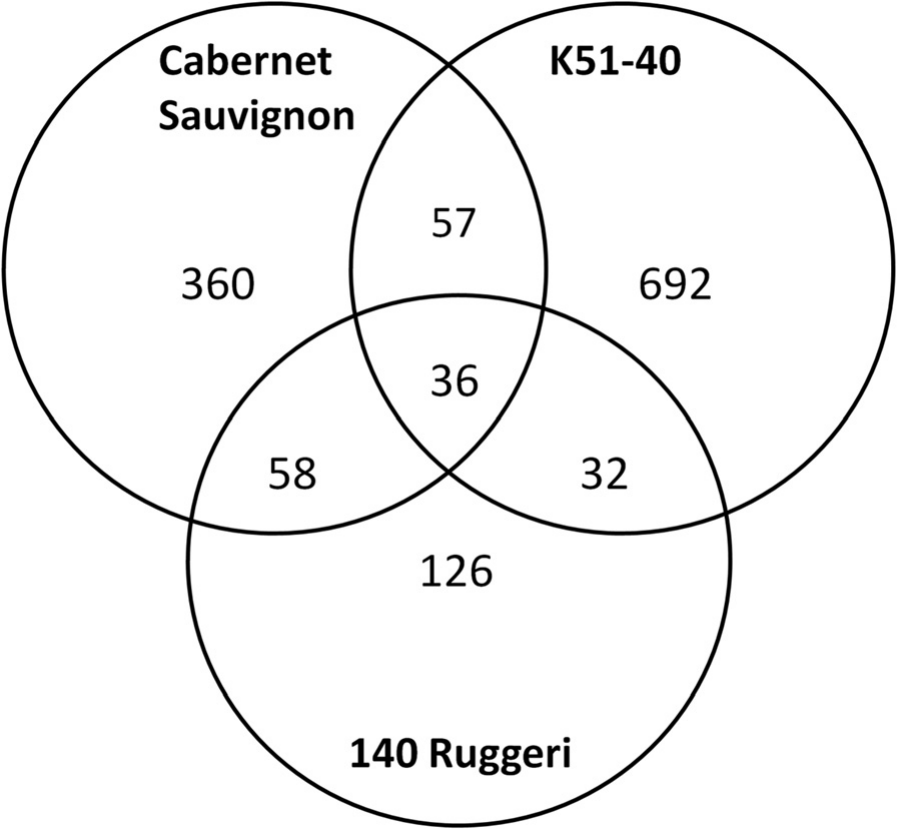
**Transcriptomic response of** ***Vitis*** **spp. to 50 mM Cl** ^**−**^ **treatment.** Venn diagram showing the number of significantly differentially expressed unique transcripts predicted by the 12xV1 annotation of the *V. vinifera* genome in Cabernet Sauvignon, 140 Ruggeri and K51-40 roots under 50 mM Cl^−^ stress. Significance was determined as *P* <0.05, ≥1.41-fold change.

Following Cl^−^ stress 1361 unique genes were differentially expressed in at least one grapevine variety (Figure 2, Additional file 6). The number of differentially expressed genes due to Cl^−^ treatment was positively correlated with Cl^−^ accumulation in shoot tissues. The Cl^−^ accumulator K51-40 had the highest number of Cl^−^ responsive transcripts (817), followed by the intermediate accumulator Cabernet Sauvignon (511), while the Cl^−^ excluder 140 Ruggeri had the least number of Cl^−^ responsive transcripts (252) (Figure 2). This correlation is consistent with findings in *Citrus* leaves when salt tolerant and sensitive rootstocks were compared after salt stress [21].

### Cluster analysis

**Figure 3:**
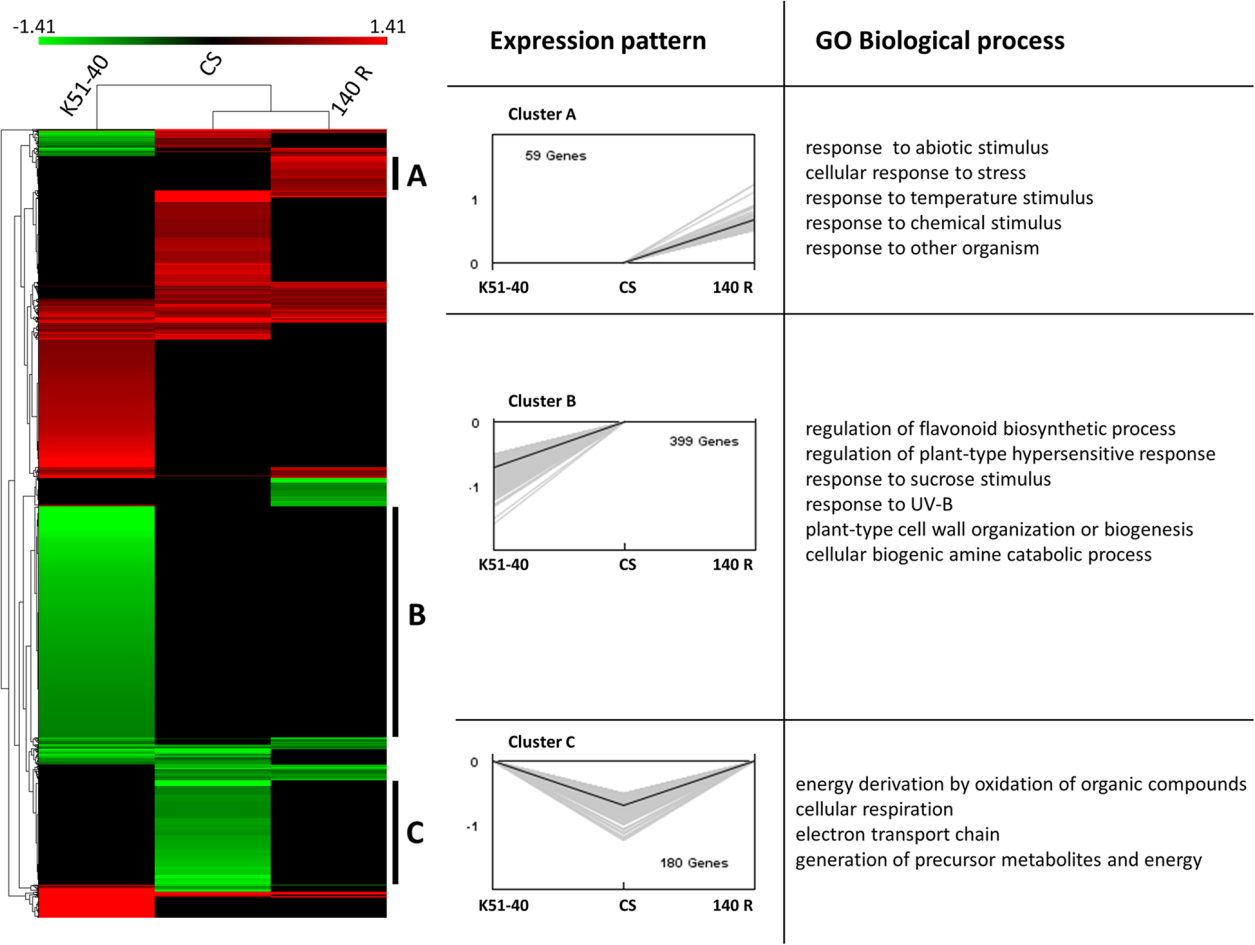
**Hierarchical clustering of chloride responsive transcripts in grapevine roots.** Transcripts (rows) that changed in response to 50 mM Cl^−^ in at least one variety with a fold change ≥ ±1.41 (*P* <0.05) were clustered. The response of each grapevine variety (columns) was also grouped (dendrogram above). Log(2) fold changes not statistically significant were set to 0. Clusters of interest are shown to the right of the heatmap, and contain genes that responded uniquely in each variety **(A, B and C)**. Expression profiles and enriched GO biological processes for each cluster are also shown to the right of the heat map. CS = Cabernet Sauvignon, 140 R =140 Ruggeri.

The transcript profiles of Cabernet Sauvignon, 140 Ruggeri and K51-40 roots exposed to high Cl^−^ were grouped by hierarchical clustering (Figure 3). 140 Ruggeri and Cabernet Sauvignon had the most similar transcriptional response to Cl^−^ in roots, while the Cl^−^ includer K51-40 had a unique response (Figure 3, top dendrogram). Gene clusters were examined by singular enrichment analysis (SEA) of gene ontology (GO) terms. Three clusters of interest showed enrichment of GO biological processes (Figure 3). Other gene clusters showed no significant enrichment of GO terms.

In 140 Ruggeri, Cl^−^ treatment induced the expression of transcripts involved in abiotic stress tolerance (Figure 3, Cluster A), including glutathione-S-transferases (GST) and heat shock proteins (HSP) (Additional file 7). Overexpression of GSTs in tobacco enhances growth under salt stress [50], while HSPs act as molecular chaperones that help maintain correct protein conformation under abiotic stress [51]. These unique trancriptional changes might enable 140 Ruggeri to perform better under salt stress relative to other grapevine genotypes.

In K51-40, Cl^−^ treatment repressed genes involved in the hypersensitive response and flavonoid biosynthesis (Figure 3, Cluster B; Additional file 8). Flavonoids have diverse roles in plants including scavenging of reactive oxygen species (ROS) and pathogen defence [52]. Under salt stress, leakage of photosynthetic and respiratory electrons may react with oxygen, leading to ROS production and subsequent oxidative stress [53]. Therefore the transcriptional regulation of flavonoid biosynthesis in K51-40 might prevent damage from excessive ROS production. In Cabernet Sauvignon, Cl^−^ treatment repressed mitochondrial specific transcripts, such as NADH dehydrogenases, c-type cytochromes and pentatricopeptide repeat (PPR) domain proteins (Figure 3, Cluster C; Additional file 9). Transcriptional repression of respiratory transcripts in Cabernet Sauvignon probably functions to reduce ROS production.

The stress-inducible phytohormone ABA restricts anion entry to the root xylem [18] and inward anion currents (anion efflux) from xylem parenchyma protoplasts from barley [17] and maize [19]. We therefore investigated whether high Cl^−^ treatment reduces the expression of genes likely to facilitate Cl^−^ transport to aerial tissues of 140 Ruggeri. Only four membrane transporters were repressed in 140 Ruggeri upon Cl^−^ treatment and none were predicted to facilitate anion movement across membranes (Additional file 10).

### Transcriptional differences between grapevine varieties under control conditions

Given that Cl^−^ accumulation in shoot tissues was significantly different between grapevine varieties in the absence of salt stress (Figure 1B and C), we hypothesised that there might be a difference in gene expression of anion transporters under control conditions. Under these conditions, 4527 genes were differentially expressed between 140 Ruggeri and K51-40 with approximately half (2310 genes) being lower in 140 Ruggeri (Additional file 11). Genes encoding 214 membrane integral proteins were expressed differently between roots of K51-40 and 140 Ruggeri (Additional file 12). Multigene families have been proposed as regulators of salt tolerance and anion homeostasis in plants, including *NRT*, *ALMT*, *SLAH* and *CLC* [9,54]. Members from these and other gene families encoding membrane proteins, as well as possible regulatory proteins, that were expressed differently between rootstocks, are summarised (Table 1) and described below. As an alternative analysis, genes with a high B-statistic (log-odds) for differential expression between rootstocks are listed in Table 2.

**Table 1:** **Differentially expressed genes between contrasting rootstocks encoding putative solute transporters under control conditions**

**Probe ID**	**12xV1 blast hit**	**12xV0 gene ID**	**Arabidopsis homolog**	**Log(2) FC 140R - K51-40**	**p-value**	**Functional annotation**
CUST_15333_17284	VIT_02s0012g01270	GSVIVT01013161001	AT4G17870	1.41	1.11E-09	Abscisic acid receptor PYL1 RCAR11
NG2_36172_20391	VIT_06s0080g00170	GSVIVT01036162001	AT1G08440	−0.69	3.02E-04	Aluminum activated malate transporter 1
CUST_44694_7793	VIT_06s0009g00450	GSVIVT01037570001	AT1G08440	0.78	1.90E-03	Aluminum activated malate transporter 1
CUST_46237_21897	VIT_08s0105g00250	GSVIVT01011148001	AT3G11680	1.30	2.51E-04	Aluminum activated malate transporter 1
CUST_8680_62299	VIT_11s0052g00320	GSVIVT01029283001	AT4G29900	−0.70	3.31E-04	Calcium ATPase 10 (ACA10), plasma membrane
NG2_12175_47390	VIT_07s0129g00180	GSVIVT01000123001	AT4G37640	0.58	8.86E-03	Calcium ATPase 2 (ACA2), plasma membrane
CUST_16133_33172	VIT_07s0129g00110	GSVIVT01000116001	AT4G37640	0.66	1.65E-02	Calcium ATPase 2 (ACA2), plasma membrane
NG2_35892_10569	VIT_06s0004g06570	GSVIVT01024741001	AT3G51860	1.44	5.00E-12	Calcium/proton exchanger CAX3
CUST_50946_56104	VIT_02s0025g04520	GSVIVT01019868001	AT1G12580	0.70	2.43E-05	Calcium-dependent protein kinase 1 CPK protein kinase
CUST_17465_49753	VIT_08s0032g00780	GSVIVT01022524001	AT2G38910	0.52	2.67E-02	Calcium-dependent protein kinase 20 CPK20
CUST_25785_57840	VIT_18s0001g00980	GSVIVT01008747001	-	−0.61	7.21E-03	Calcium-dependent protein kinase 9 CPK9
CUST_45042_37341	VIT_15s0021g01150	GSVIVT01018316001	AT1G28710	−1.59	4.74E-07	Calcium-dependent protein kinase-related
CUST_46046_19308	VIT_01s0010g02150	GSVIVT01010291001	AT1G14590	1.08	3.25E-07	Calcium-dependent protein kinase-related
CUST_25533_22696	VIT_05s0020g04240	GSVIVT01018059001	AT5G57110	0.76	7.26E-04	Calcium ATPase 12 (ACA12)
CUST_38995_37629	VIT_14s0030g02090	GSVIVT01021803001	AT3G63380	1.41	2.60E-05	Calcium ATPase 12 (ACA12)
CUST_40093_46251	VIT_05s0020g04260	GSVIVT01018061001	AT3G22910	−0.63	9.81E-03	Calcium ATPase 13 (ACA13)
NG2_7370_1539	VIT_09s0018g01840	GSVIVT01016118001	AT3G13320	0.99	1.10E-05	Cation exchanger (CAX2)
CUST_43832_58554	VIT_08s0056g01480	GSVIVT01029961001	AT5G17860	0.97	4.47E-03	Cation exchanger (CAX7)
NG11_49713_18843	VIT_14s0068g02190	GSVIVT01033108001	AT3G27170	−0.61	7.55E-05	Chloride channel B (CLC-b)
NG11_46088_11883	VIT_19s0015g01850	GSVIVT01014852001	AT1G55620	1.37	2.85E-34	Chloride channel F (CLC-f)
NG11_51750_10097	VIT_06s0004g03520	GSVIVT01025107001	AT3G45650	1.27	7.60E-19	Nitrate excretion transporter 1
NG11_44542_25973	VIT_06s0004g03530	GSVIVT01025106001	AT3G45650	1.61	1.24E-32	Nitrate excretion transporter 2
NG11_46422_21127	VIT_11s0016g05170	GSVIVT01015522001	AT2G26690	−1.22	2.58E-19	Nitrate transporter 1.4
CUST_37073_22417	VIT_01s0127g00070	GSVIVT01013802001	AT1G12940	0.63	6.29E-03	Nitrate transporter 2.5
CUST_42271_1540	VIT_14s0066g00850	GSVIVT01032430001	AT5G14570	1.59	8.82E-06	Nitrate transporter 2.7
NG2_12101_30038	VIT_03s0097g00510	GSVIVT01038513001	AT5G64410	0.87	1.65E-06	Oligopeptide transporter OPT4
NG12_21396_16431	VIT_12s0035g01820	GSVIVT01023146001	AT1G59740	0.52	2.47E-05	Proton-dependent oligopeptide transport (POT) family protein
NG11_4749_12704	VIT_17s0000g05550	GSVIVT01008072001	AT3G47960	0.54	1.88E-04	Glucosinolate transporter 1 (GTR1)
NG11_7897_10153	VIT_14s0066g02020	GSVIVT01032550001	AT5G14940	0.64	3.31E-07	Proton-dependent oligopeptide transport (POT) family protein
NG11_35177_1429	VIT_18s0041g00670	GSVIVT01026058001	AT1G72140	0.89	7.91E-14	Proton-dependent oligopeptide transport (POT) family protein
NG11_25530_14040	VIT_04s0008g03580	GSVIVT01035643001	AT1G22550	1.11	2.21E-24	Nitrate transporter 1.11
NG11_31776_20297	VIT_16s0050g01860	GSVIVT01028789001	AT5G24030	0.54	4.61E-05	SLAH3 (SLAC1 Homologue 3)
CUST_21950_56777	VIT_07s0191g00070	GSVIVT01003419001	AT4G40010	−1.01	1.28E-04	SNF1-related protein kinase 2.7 (SnRK2.7)
CUST_41758_42394	VIT_00s0710g00020	GSVIVT01002389001	AT4G33950	−0.56	1.54E-02	SNF1-related protein kinase 2.6 (SnRK2.6)
CUST_27252_1533	VIT_01s0011g06550	GSVIVT01011573001	AT2G01980	−2.30	1.53E-05	SOS1 (Na+/H + antiporter)
CUST_15165_41173	VIT_06s0004g07830	GSVIVT01024587001	AT5G58380	−0.73	1.72E-06	SOS2 (salt overly sensitive 2)
CUST_27642_7432	VIT_16s0098g01870	GSVIVT01038549001	AT5G24270	−0.67	8.65E-03	SOS3 (salt overly sensitive 3)

List of significantly differentially expressed genes (*P* <0.05, ≥ ±1.41 fold) between the contrasting grapevine rootstocks 140 Ruggeri and K51-40 in the absence of Cl^−^ treatment that have putative roles in ion homeostasis. Positive log(2) FC values = higher in 140 Ruggeri.

**Table 2:** **Highly significantly differentially expressed genes between contrasting rootstocks under control conditions**

**Probe ID**	**12xV1 blast hit**	**12xV0 gene ID**	**Arabidopsis homolog**	**Log2 FC 140 R - K51-40**	**p-value**	**B**	**Functional annotation**
NG11_47168_24630	VIT_09s0002g02430	GSVIVT01016879001	AT3G21250	−1.90	1.69E-42	89.62	ABC transporter C member 12
NG11_46088_11883	VIT_19s0015g01850	GSVIVT01014852001	AT1G55620	1.37	2.85E-34	70.44	CLCf (chloride channel F)
NG11_44542_25973	VIT_06s0004g03530	GSVIVT01025106001	AT3G45650	1.61	1.24E-32	66.55	Nitrate excretion transporter 2
NG2_21308_18913	VIT_11s0016g02570	GSVIVT01015240001	AT2G19690	3.01	1.05E-26	56.42	Phospholipase A2 precursor
NG2_12381_40127	VIT_06s0004g06340	GSVIVT01024768001	AT5G58800	3.35	5.46E-26	54.67	Flavodoxin-like quinone reductase 1
NG2_21123_37199	VIT_12s0028g02740	GSVIVT01020642001	-	−4.06	1.36E-24	51.28	Isoflavone methyltransferase/Orcinol O-methyltransferase 1 oomt1
NG11_25530_14040	VIT_04s0008g03580	GSVIVT01035643001	-	1.11	2.21E-24	47.44	Nitrate transporter 1.11
NG2_12165_35517	VIT_13s0073g00250	GSVIVT01034634001	AT2G26230	−5.07	9.34E-23	46.84	Urate oxidase
NG2_48691_28703	VIT_15s0046g01950	GSVIVT01026987001	-	2.97	4.01E-22	45.28	Anthocyanidine rhamnosyl-transferase
NG2_28672_23579	VIT_10s0003g03780	GSVIVT01021513001	AT1G30130	2.18	7.23E-22	44.65	Unknown protein
NG2_35994_23405	VIT_18s0001g13850	GSVIVT01009855001	AT4G31500	−3.20	1.93E-21	43.62	Cytochrome P450, family 83, subfamily B, polypeptide 1
NG2_48494_21157	VIT_18s0001g13820	GSVIVT01009854001	AT4G31500	−3.32	1.78E-19	38.90	Cytochrome P450, family 83, subfamily B, polypeptide 1
NG2_5431_23220	VIT_00s0153g00040	GSVIVT01001251001	-	−2.73	1.90E-19	38.83	S-locus receptor kinase
NG2_48249_3223	VIT_03s0038g01760	GSVIVT01024088001	-	3.48	2.76E-19	38.44	Disease resistance protein (CC-NBS class)
NG2_33320_2332	VIT_08s0007g01590	GSVIVT01034034001	-	−1.80	5.70E-19	37.65	Fructose 1,6-bisphosphatase
NG2_21199_29690	VIT_06s0004g00730	GSVIVT01025431001	AT3G13550	2.00	8.19E-19	37.26	Ubiquitin-conjugating enzyme E2 D/E
NG2_11819_5360	VIT_05s0094g00120	GSVIVT01038099001	AT3G59600	2.61	3.10E-18	35.84	DNA-directed RNA polymerase II subunit H
NG11_46422_21127	VIT_11s0016g05170	GSVIVT01015522001	AT2G26690	−1.22	2.58E-19	35.59	Nitrate transporter 1.4
NG2_12023_10427	VIT_18s0001g05430	GSVIVT01036371001	-	3.07	4.99E-18	35.33	(+)-delta-cadinene synthase isozyme XC14
NG2_45557_29139	VIT_10s0042g01130	GSVIVT01026257001	AT4G19670	3.69	7.83E-18	34.85	Zinc finger (C3HC4-type ring finger)
NG2_40716_2133	VIT_06s0061g00120	GSVIVT01031543001	-	3.17	7.99E-18	34.82	Beta-1,3-glucanase [*Vitis riparia*]
NG11_51750_10097	VIT_06s0004g03520	GSVIVT01025107001	AT3G45650	1.27	7.60E-19	34.49	Nitrate excretion transporter 1
NG2_45497_36221	VIT_12s0028g02810	GSVIVT01020636001	-	−1.58	2.64E-17	33.55	Isoflavone methyltransferase/Orcinol O-methyltransferase 1 oomt1
NG2_6989_23958	VIT_06s0004g05440	GSVIVT01024878001	AT2G29260	−1.23	3.46E-17	33.27	Tropinone reductase
NG2_5127_34182	VIT_03s0097g00620	GSVIVT01038529001	AT5G64440	1.71	4.59E-17	32.98	N-acylethanolamine amidohydrolase
NG2_7581_45053	VIT_06s0080g00800	GSVIVT01036089001	AT5G22360	2.01	7.43E-17	32.47	Vesicle-associated membrane protein 714
NG2_12628_32903	VIT_10s0071g00440	GSVIVT01034406001	AT4G11900	−3.24	7.90E-17	32.40	Serine/threonine-protein kinase receptor ARK3
NG2_48742_21119	VIT_08s0007g09030	GSVIVT01033230001	-	−1.40	2.03E-16	31.39	DnaJ homolog, subfamily A, member 5
NG2_575_20076	VIT_16s0098g01670	GSVIVT01038570001	AT5G53070	1.57	3.23E-16	30.90	Ribosomal protein L9
NG2_5167_10137	VIT_05s0029g00770	GSVIVT01020981001	-	−1.67	3.40E-16	30.84	Nematode resistance-like protein
NG2_12777_24696	VIT_18s0117g00080	GSVIVT01012796001	AT5G36930	3.34	3.95E-16	30.68	R protein L6
NG2_5559_11115	VIT_02s0025g00930	GSVIVT01019469001	AT3G59140	−1.63	5.03E-16	30.43	Multidrug resistance-associated protein 14
NG2_36555_51297	VIT_03s0088g00390	GSVIVT01037045001	AT5G23590	1.34	6.02E-16	30.24	DnaJ homolog, subfamily C, member 17

Highly significantly differentially expressed unique genes (*P* <0.05, ≥ ±1.41 fold, B >30) between 140 Ruggeri and K51-40 root tissue under control conditions identified using the B-statistic. Positive log(2) FC values = higher in 140 Ruggeri.

### ***NRT***/***POT***

**Figure 4:**
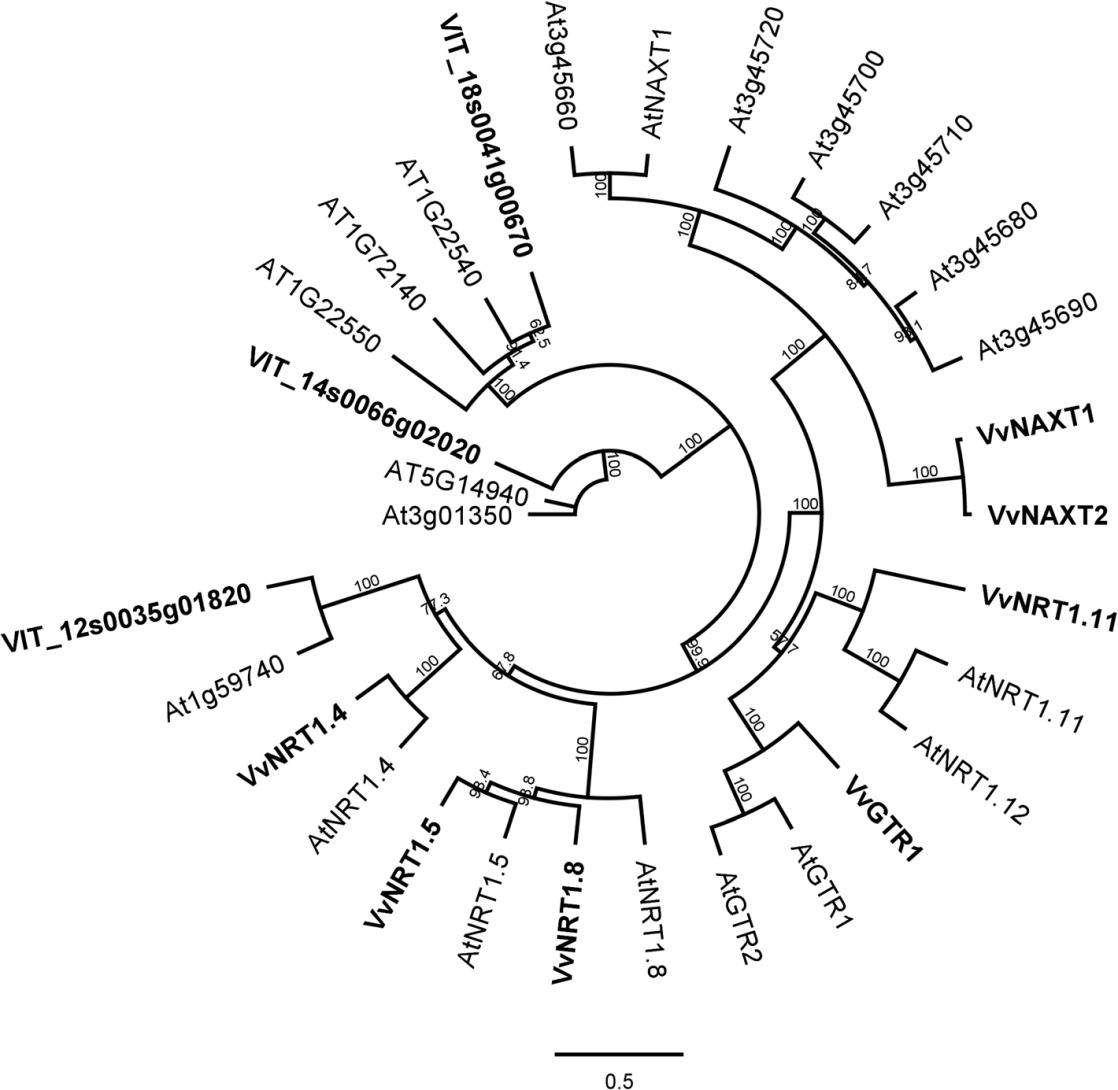
**Phylogenetic relationship between Arabidopsis and grapevine** ***NRT/POT*** **gene family members.** Unrooted neighbour-joining tree of Arabidopsis and grapevine (bold) *NRT/POT* family members with bootstrap values from 1000 iterations. Scale = substitutions per site. Gene identifiers for the protein sequences used are shown in Additional file 2, while the multiple sequence alignment is shown in Additional file 3.

The NRT or proton dependent oligopeptide (POT) gene family is involved in the acquisition and whole plant homeostasis of nitrogen; different family members transport NO_3_^−^, amino acids and various peptides [55]. In our study, 8 *NRT1* genes were expressed differently between rootstocks (Table 1). Grapevine *NRT1* gene family members were poorly annotated in functional databases. To assign putative functions, we produced a phylogeny of the grapevine *NRT*s uncovered in our microarray screen using Arabidopsis *NRT1*s. Homologs of *AtNRT1.4*, *AtNRT1.11*, *nitrate excretion transporter 1 (AtNAXT1), AtNAXT2* and *glucosinolate transporter 1* (*AtGTR1)* were identified, as well as three other *Vitis NRT*s with uncharacterised Arabidopsis homologs (Figure 4). Two grapevine *NRT*s homologous to Arabidopsis *AtNRT2.5* and *AtNRT2.7*, as well as a homolog of Arabidopsis oligopeptide transporter 4 (OPT4) were more abundantly expressed in 140 Ruggeri (Table 1). Differential expression of *VvNAXT1*, *VvNAXT2*, *VvNRT1.11* (all higher in 140 Ruggeri) and *VvNRT1.4* (higher in K51-40) was also highly significant (Table 2).

**Figure 5:**
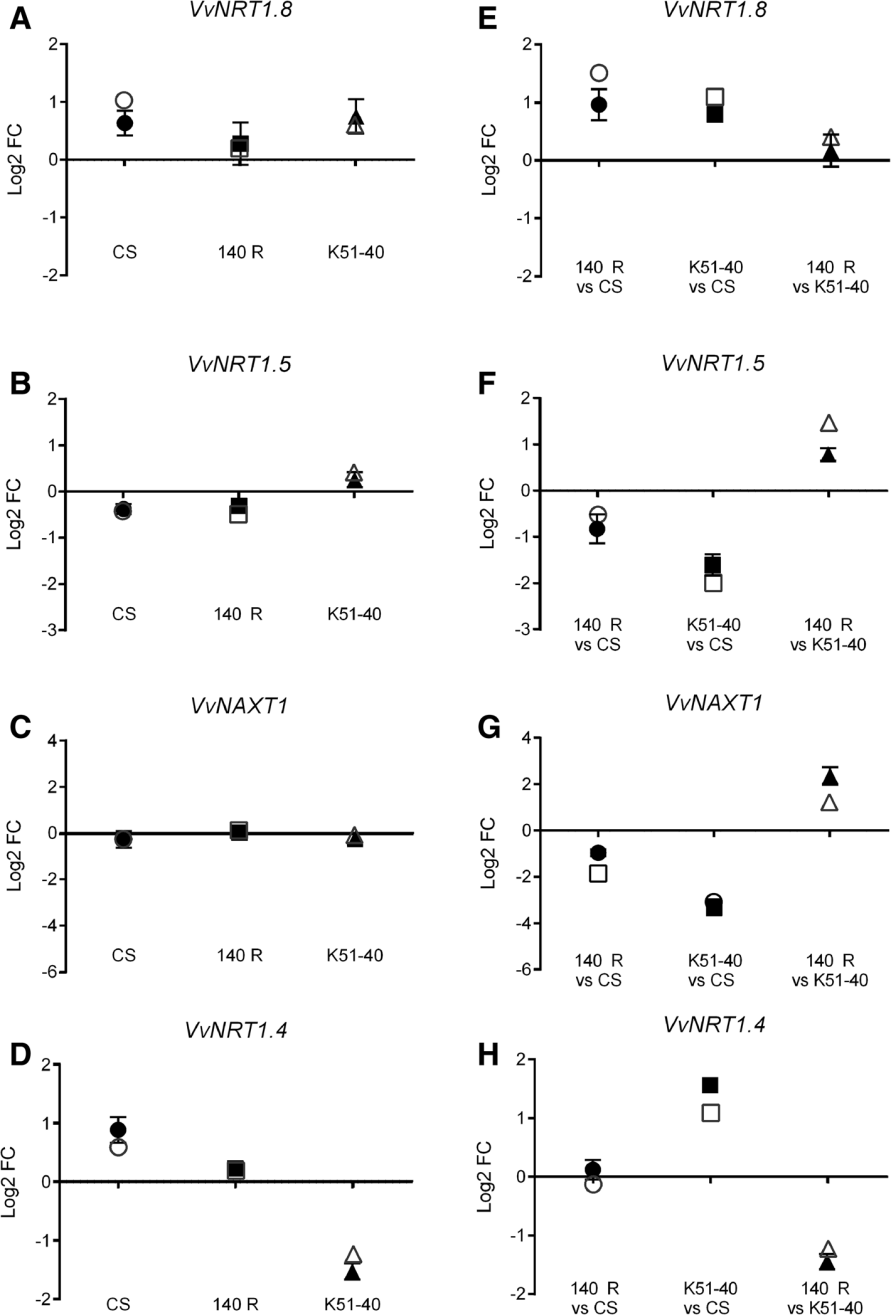
**mRNA expression changes of four** ***Vitis NRT1*** **family members in three grapevine genotypes under salt stress and control conditions. (A – D)** Log2 mRNA fold changes of *VvNRT1.8* **(A)** *VvNRT1.5* **(B)** *VvNAXT1* **(C)** *VvNRT1.4* **(D)** in response to 50 mM Cl^−^ treatment as determined by qRT-PCR (filled symbols) and microarray hybridisation (open symbols). **(E – H)** Log2 mRNA fold differences of *VvNRT1.8* **(E)** *VvNRT1.5* **(F)** *VvNAXT1* **(G)** *VvNRT1.4* **(H)** between grapevine genotypes under control conditions as determined by qRT-PCR (filled symbols) and microarray hybridisation (open symbols). For qRT-PCR data points, the bars represent the mean ± SEM of four biological replicates. CS = Cabernet Sauvignon, 140 R = 140 Ruggeri. The E-value of *VvNRT1.5* probe is above the threshold used for all other probes analysed in this study.

**Figure 6:**
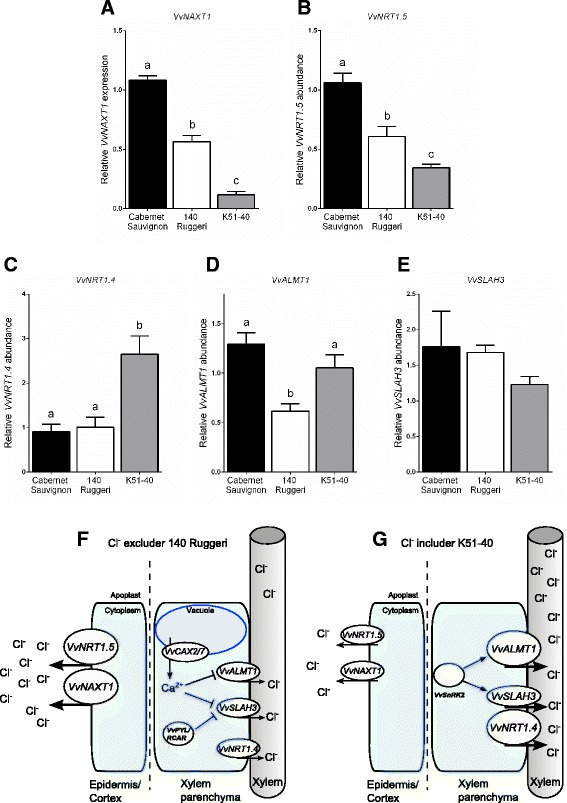
**Relative transcript abundances of membrane proteins in roots of grapevine genotypes under control conditions measured by qRT-PCR, and a model indicating possible molecular mechanisms for reduced net xylem loading of Cl** ^**−**^ **in 140 Ruggeri. (A – B)** relative expression levels of *VvNAXT1* **(A)** and *VvNRT1.5* **(B)** measured by qRT-PCR, which represent possible avenues for cortical or epidermal efflux of Cl^−^ out of roots. **(C – E)** relative expression levels of *VvNRT1.4* **(C)**, *VvALMT1* **(D)** and *VvSLAH3* **(E)** measured by qRT-PCR, which represent possible avenues for xylem loading of Cl^−^. Bars represent the mean of four biological replicates ± SEM. Transcript abundance is relative to the Cabernet Sauvignon biological replicate with the lowest cycle threshold (Ct) value, which was set to 1. Statistical differences were determined using one way ANOVA with Holm-Sidak's multiple comparisons test to compare the means. **(F - G)** proposed model for reduced net xylem loading of Cl^−^ in 140 Ruggeri relative to K51-40. **(F)** In 140 Ruggeri, anion efflux from cortical or epidermal root cells could be mediated through putative anion channels VvNRT1.5 and VvNAXT1 which are transcriptionally more abundant in the Cl^−^ excluder. Xylem loading of Cl^−^ could be restricted through reduced VvNRT1.4 abundance, or inhibition of VvSLAH3 and VvALMT by higher [Ca^2+^]_cyt_ mediated by VvCAX3 (directly, or in partnership with Ca^2+^ dependent protein kinases). **(G)** In K51-40, anion efflux to the xylem apoplast could be enhanced through increased abundance of VvALMT1 and VvNRT1.4, and activation of VvALMT1 and VvSLAH3 by SnRK2 kinases.

In Arabidopsis roots, *AtNRT1.8* is induced and *AtNRT1.5* repressed by salt and cadmium stress [56]. AtNRT1.5 is the only NRT1 isoform with a confirmed role in root xylem loading of NO_3_^−^ [57], and mutants of *atnrt1.5* grow better under NaCl stress than wildtype [58]. Conversely, AtNAXT1 effluxes NO_3_^−^ under acid load, and is regulated at the post-transcriptional level [59]. We further investigated expression patterns of *Vitis* orthologs of these genes. *VvNRT1.8* and *VvNRT1.5* were identified phylogenetically (Figure 4). They were oppositely regulated by salt stress in Cabernet Sauvignon and 140 Ruggeri, but not K51-40, although the expression changes were small (Figure 5A and B). *VvNAXT1* was unresponsive to salt in all three genotypes (Figure 5C), which is consistent with the response of its homolog in Arabidopsis [59]. Interestingly, *VvNRT1.4* was strongly repressed (3 fold) by salt stress in K51-40 (Figure 5D). In spite of these differences in salt response, the largest transcriptional differences in grapevine *NRT1* mRNAs were observed between genotypes under control conditions, especially between the contrasting rootstocks 140 Ruggeri and K51-40 (Figure 5E – H). This suggests of a role of some of these genes in Cl^−^ exclusion in the absence of stress (Figure 6). Arabidopsis *AtNRT1.5* is considered important for plant salt tolerance [58], possibly due a role in anion loading to the xylem [57]. In grapevine, *VvNRT1.5* was not preferentially expressed in the root stele under salt stress (Additional file 13), which contrasts with *AtNRT1.5* [57]. Furthermore, *VvNRT1.5* was more abundant in 140 Ruggeri than K51-40 (Figure 5F; Figure 6B). These data reduce the likelihood of VvNRT1.5 having a role in xylem loading of Cl^−^ in grapevine. Based on transcriptional data, we suggest that *VvNRT1.4* is the best *NRT1* candidate for xylem loading of Cl^−^ due to a much greater abundance in K51-40 roots under control conditions (Figure 5D; Figure 6C).

### ALMT

Chelation of toxic aluminium in the rhizosphere by the efflux of organic acids from roots is facilitated by plasma membrane aluminium-activated malate transporters (ALMT) [60]. ALMTs are a large multigene family with multiple roles; despite their name most ALMTs are not activated by aluminium and they allow the permeation of various anions. For example, ALMTs function in anion homeostasis and mineral nutrition, (ZmALMT1) [61], or Cl^−^ transport across the tonoplast (AtALMT9) [62]. Root ALMTs might therefore have a role in Cl^−^ exclusion. Three *ALMT1* homologs were differentially expressed between rootstocks (Table 1). Whether these proteins mediate Cl^−^ fluxes, and the directionality of such fluxes, remains unresolved, but Cl^−^ exclusion could arise through efflux of Cl^−^ to the rhizosphere (higher expression in 140 Ruggeri, *VIT_06s0009g00450*, *VIT_08s0105g00250*) (Table 1) or reduced Cl^−^ entry in the cortex and restricted xylem loading of Cl^−^ (lower expression in 140 Ruggeri, *VIT_06s0080g00170*) (Table 1; Figure 6D and G).

### Calcium transporters (CAX and ACA)

Calcium exchangers (CAX) mediate Ca^2+^/cation antiport activity across the tonoplast. Roles of CAXs include cell specific storage of Ca^2+^ by CAX1 [63], while Arabidopsis *cax3* mutants are sensitive to NaCl, LiCl and acidic pH, suggesting a possible role in salt tolerance [64]. Three grapevine *CAX* transcripts were more abundant in roots of 140 Ruggeri compared to K51-40 (Table 1). In addition to CAX, the plant plasma and vacuolar membranes harbour auto-inhibited Ca^2+^-ATPases (ACA), of which Arabidopsis *ACA4* can improve salt tolerance of yeast [65]. Six *ACA*s were differentially expressed between 140 Ruggeri and K51-40. These data indicate that genes regulating cytosolic free calcium ([Ca^2+^]_cyt_) in roots could be important for grapevine Cl^−^ exclusion.

### CLC

Two CLCs showed differential expression between rootstocks under control conditions. A gene homologous to Arabidopsis *AtCLCb* (*VIT_14s0068g02190*) was less abundant in 140 Ruggeri (Table 1). Another *CLC* with homology to *AtCLCf* (*VIT_19s0015g01850*) was less abundant in K51-40 (Table 1). Differential expression of *VvCLCf* was also identified as highly statistically significant (Table 2).

### SLAH3 and ABA signalling

Homologs of the Arabidopsis SLAC1 anion channel (AtSLAH1 and AtSLAH3) are plasma membrane localized, expressed in the root vasculature, and functionally complement guard cell anion efflux in the *slac1* mutant [23]. This indicates that SLAHs might be involved in anion homeostasis [23] and loading to the xylem sap [54]. *VvSLAH3* was more abundant in the Cl^−^ excluder 140 Ruggeri compared to K51-40 under control conditions (Table 1; Figure 6E). This contrasts with *Citrus*, where *CcSLAH1* was up-regulated by 90 mM salt stress in a Cl^−^ accumulating rootstock [22]. Reconstitution in *X. laevis* oocytes has demonstrated that plant SLAC/SLAH activity is tightly regulated by kinase/phosphatase activity following an ABA signal [66]. Homologs of the Arabidopsis ABA signalling machinery were differentially expressed between rootstocks. The ABA receptor *VvPYL1/RCAR11* was more highly expressed in 140 Ruggeri (Table 1). Two SNF1-related protein kinase 2 *(SnRK2)* family members including the *Vitis* ortholog of *SnRK2.6* (open stomata 1 (OST1)) were repressed in 140 Ruggeri, and multiple calcium dependent protein kinases (*CPK*) were differentially expressed between rootstocks (Table 1). Homologs of these genes in other plants have proven roles in ABA induced activation of SLAC1 in guard cells [67] and might be involved in SLAH3 regulation in *Vitis* roots.

### Other candidates

Two ABC transporters were significantly differentially expressed between rootstocks; a C-type (*ABCC 12*) (*VIT_09s0002g02430*) (higher in 140 Ruggeri) and multidrug resistance 12-type (*VIT_02s0025g00930*) (higher in K51-40) (Table 2). A role in Cl^−^ transport has not been identified for ABC transporters in plants, although reports suggest roles in arsenic tolerance [68] and salt tolerance [69]. A C3HC4-type ring finger protein was more abundant in the tolerant variety 140 Ruggeri (Table 2). A C3HC4 protein was potentially crucial for abiotic stress tolerance in rice roots [70]. A phospholipase A2 precursor (*VIT_11s0016g02570*) was also expressed alternatively between rootstocks (Table 2). The product of a phospholipase A2 activates a tonoplast H^+^/Na^+^ antiporter in cultured cells of California poppy [71].

## Discussion

Shoot chloride exclusion is one of several traits that underpins salt tolerance. However, the root-localised anion transport proteins (or their regulators) thought to be crucial for salt tolerance remain unidentified [9,15,16,22]. We therefore analysed the genome wide transcriptional response of grapevine roots to Cl^−^ stress. Cabernet Sauvignon repressed transcripts encoding respiratory proteins, probably to reduce ROS production under salt stress. Although ROS may act as signalling molecules in eukaryotes [72,73], it has previously been reported that ROS production in grapevine cells that contrast in salt-tolerance represents a manifestation of cellular damage rather than an adaptive response [74]. We therefore propose a hierarchy exists in the magnitude of transcriptional responses to Cl^−^ stress (K5140> > Cabernet Sauvignon> > 140 Ruggeri) that correlates with the amount of damage in the laminae. However, these differences in varietal responses to stress do not explain differential Cl^−^ exclusion, which was statistically significant before salt stress (Figure 1).

Studies indicate that there is natural variation in the ability to tolerate salt stress in various plant species including *Citrus* [22], rice [70], barley [75,76], and Arabidopsis [77]. Our data support the hypothesis that Cl^−^ exclusion in grapevine is mediated by anion transporters or channels that are differentially expressed between non-stressed 140 Ruggeri and K51-40. To this end, we have proposed a testable model for Cl^−^ exclusion based on the expression levels of candidate genes identified in our study (Figure 6). These candidate genes for Cl^−^ exclusion are subsequently discussed in the context of this model and existing literature.

In plant roots, anion movement across the plasma membrane of xylem parenchyma cells for loading to xylem vessels occurs through unidentified anion channels with fast and slow activation kinetics (X-QUAC and X-SLAC respectively) [17]. Anion conductances in Arabidopsis guard cells with homologous activation kinetics have been characterised, and the channels eliciting these currents identified. The slowly activating anion conductance in guard cells has been attributed to AtSLAC1 and AtSLAH3 channels [24,78], while guard cell QUAC is mediated by AtALMT12 [79]. It is therefore feasible that X-SLAC and X-QUAC arise from SLAH and ALMT channels in root cells. We identified *VvSLAH3* and three *VvALMT1* transcripts that were expressed differently between rootstocks. Thermodynamics predicts that the loading of Cl^−^ into the xylem under low apoplastic [Cl^−^] occurs by passive transport [80]. Therefore, transcripts that encode putative anion transport proteins with a high abundance in K51-40 are good candidates for controlling xylem loading of Cl^−^. Our results suggest that VvALMT1 may be involved in xylem loading of Cl^−^ (Figure 6D and G). *VvSLAH3* transcript was more abundant in 140 Ruggeri (Table 1; Figure 6E). For it to be involved in xylem loading of Cl^−^ there are two alternatives. Arabidopsis SLAH3 has been shown to be much more permeable for NO_3_^−^ than Cl^−^ [78]. If this is the case in grapevine, the pathway for anion transport in 140 Ruggeri could be more NO_3_^−^ selective than in K51-40, thus resulting in greater discrimination against Cl^−^ loading of the xylem in 140 Ruggeri. Alternatively, SLAH3 could be permeable to Cl^−^ but the extent of post-translational control differs between varieties, as elaborated below.

Cellular anion conductance must be tightly regulated to avoid uncontrolled electrolyte efflux [54], and for this reason complex signalling networks exist in plants. Upon an ABA induced rise in [Ca^2+^]_cyt_, guard cell SLAC may be activated by calcium dependent protein kinases CPK23 and CPK21 [67]. Alternatively, the Ca^2+^ independent kinase SnRK2.6 (OST1) can activate both guard cell SLAC and QUAC in response to ABA [81]. In contrast, opposite regulation by ABA and [Ca^2+^]_cyt_ occurs in root cells, with X-QUAC being inhibited by ABA and by high [Ca^2+^]_cyt_ [17,19]; whether kinases are involved in this regulation have not yet been determined. A transcript encoding the *VvPYL1/RCAR11* ABA receptor was significantly more abundant in roots of 140 Ruggeri compared to K51-40. The Cl^−^ excluder might therefore be more sensitive to ABA, or may be primed for any slight increase in ABA concentration. Differential expression of vacuolar *CAXs* and *ACAs* between rootstocks might function to maintain [Ca^2+^]_cyt_ signals in root cells of 140 Ruggeri, thus participating in the Ca^2+^ dependent down-regulation of X-QUAC and X-SLAC (Figure 6F). AtCPK20 interacts with AtSLAH3 in Arabidopsis pollen tubes [82]. Differential expression of *VvCPK20*, among other *CPKs*, between rootstocks might indicate an involvement of these kinases in *VvSLAH3* regulation. In addition, differential expression of *VvSnRK2.6* and *VvSnRK2.7* between rootstocks implicates both the Ca^2+^ dependent and independent ABA signalling machinery in grapevine roots as possible mediators of Cl^−^ exclusion (Figure 6G). The sheer number of genes potentially involved in X-QUAC and X-SLAC mediated pathways, possible kinase redundancy or multiple kinase targets, could explain the observations that Cl^−^ exclusion in grapevine is polygenic [15,83].

Arabidopsis has 53 *NRT1* genes and rice has 80 *NRT1* members. This has led to the question [55]: are there unidentified anionic substrates for NRTs beyond just nitrate or peptides to account for such large gene families? The large number of *NRT1* genes identified in our screen suggests they might play some key role in Cl^−^ homeostasis. Plasma membrane localisation of plant NRTs heightens the possibility for roles in cellular Cl^−^ fluxes. However, the anion selectivities of plant NRTs have been rarely examined [9]. AtNAXT1 was shown not to transport Cl^−^ [59], but characterisation of the remaining 6 Arabidopsis NAXTs is yet to be reported. If permeable to Cl^−^, greater abundance of *VvNAXT1* and *VvNAXT2* in 140 Ruggeri compared to K51-40 (Table 1; Table 2; Figure 6A) could allow the Cl^−^ excluding rootstock to excrete Cl^−^ back to the external medium instead of transporting it to the shoot (Figure 6F). This function might be enhanced under salinity stress if cytosolic pH is reduced, as *AtNAXT1* actively excretes anions under acid load, possibly to balance proton extrusion by H^+^-ATPases [59]. This cannot work as the sole mechanism of Cl^−^ exclusion, as 140 Ruggeri still retains more Cl^−^ in the roots compared to Cabernet Sauvignon and K51-40 (Figure 1A). Other stress responsive plant *NRT1*s (*VvNRT1.8, VvNRT1.5*) showed similar expression profiles to orthologous genes in Arabidopsis. However, *VvNRT1.5* was less abundant in the root stele compared to the cortex (Additional file 13), indicating that this gene is more likely to be involved in cortical efflux of Cl^−^ rather than Cl^−^ loading to the xylem (Figure 6F and G). Excessive Cl^−^ in the root zone or cytoplasm could inhibit NO_3_^−^ transport (both uptake and efflux) due to the well-documented antagonism between these anions [84,85]. Therefore, differences in *NRT1* expression in salt stressed grapevine roots also could be a compensatory mechanism to overcome this ionic antagonism.

Multiple studies have linked plant CLCs to salt tolerance [86-89]. *VvCLCb* and *VvCLCf* were expressed differently between rootstocks under control conditions. In Arabidopsis, *AtCLCb* is a vacuolar NO_3_^−^/H^+^ exchanger [90], as is *AtCLCa* [91]. A single missense mutation in *AtCLCa* changes the selectivity from NO_3_^−^ to Cl^−^ [92,93]. It is therefore possible that *VvCLCb* in *Vitis* roots participates in Cl^−^ sequestration in cell vacuoles, although greater expression in K51-40 does not fully support this. AtCLCf is associated with the trans-Golgi network [94], so a role in salt tolerance is less likely. Further study into *Vitis* CLCs is therefore needed before concluding a role in grapevine salt tolerance.

Candidate genes for plant Cl^−^ exclusion identified by Brumos *et al.* [22] were not highlighted in our study. Our array design had two probes for the putative Cl^−^ conductance regulator *VvICln* (*VIT_16s0022g01560*) and neither were salt responsive, consistent with short-term stress response in *Citrus* but contrasting with the long-term results [22]. On the other hand, one probe showed statistically significant differential expression between varieties under control conditions but was greater in the Cl^−^ excluding rootstock (data not shown). This means if *VvICln* contributes to Cl^−^ exclusion in 140 Ruggeri, it must act as a negative regulator of Cl^−^ conductance. This seems unlikely given data in animals and plants [22,26]. *VvCCC* and *VvSLAH1* were also not differentially expressed, and so if they are involved may be regulated at the post-translational level, which cannot be highlighted by microarray technology. Indeed the activity of many plant anion channels is modified by phosphorylation events such as AtNRT1.1 [95], AtSLAC1 [96] and AtCLCa [97]. Differences in expression of SLAC/SLAH regulators *SnRK2* and *CPK* between rootstocks ensures *VvSLAH1* remains a candidate for Cl^−^ homeostasis in *Vitis*. In future studies, it would be valuable to identify interacting partners of the protein kinases identified as differentially expressed in this study, and any functional changes induced by such interactions.

## Conclusions

Using a whole root transcriptome approach, a detailed analysis of root mRNA profiles of contrasting grapevine genotypes is presented. This provides a complement to earlier physiological studies of the same varieties that have demonstrated the mechanism of shoot Cl^−^ exclusion as the restriction of its net xylem loading at the root [15,16,98]. A valuable list of candidate genes likely to mediate shoot Cl^−^ exclusion has been identified. Future functional characterisation of these genes, including the elucidation of protein-protein interactions, may enable their use in grapevine rootstock breeding efforts. More broadly the further study of these genes and their homologs in other species will aid our understanding of long distance Cl^−^ transport in plants.

### Availability of supporting data

Data supporting the results of this article are available in the Gene Expression Omnibus repository (http://www.ncbi.nlm.nih.gov/geo/) under accession number GSE57770. An ArrayQualityMetrics report is available at http://dx.doi.org/10.6070/H4CZ354H.

## Additional files

Additional file 1:
**List of 90 predicted** ***V. vinifera*** **genes encoding putative anion transporters, and their predicted functional annotation, that were spotted onto the custom Agilent microarray slides multiple times for B-statistic analysis.**
Additional file 2:
**Gene identifiers and annotations of the amino acid sequences used in the phylogenetic analysis of interesting NRT1 members.**
Additional file 3:
**Multiple sequence alignment of Arabidopsis and grapevine NRT members for the data presented in Figure** 4 **.** Shading is representative of the Blosum62 score matrix as follows: black (100% similar) dark grey (80% similar) light grey (60% similar) unshaded (<60% similar). Additional file 4:
**List of primers used in this study.**
Additional file 5:
**Correlation between expression ratios determined by Agilent custom gene expression array and qRT-PCR.** (a) Gene expression ratios of control to salt treated samples were compared for 12 genes (*VIT_00s0229g00130, VIT_01s0011g06550, VIT_02s0012g01160, VIT_06s0004g03520, VIT_06s0080g00170, VIT_08s0040g01890, VIT_08s0040g03220, VIT_11s0016g05170, VIT_13s0019g00330, VIT_14s0108g00700, VIT_15s0021g00330, VIT_16s0050g01860*) in all 3 grapevine varieties by microarray and qRT-PCR. (b) Gene expression ratios of varietal differences under control conditions were compared for 12 genes (*VIT_01s0011g06550, VIT_02s0012g01160, VIT_06s0004g03520, VIT_06s0080g00170, VIT_08s0040g01890, VIT_08s0040g03220, VIT_11s0016g05170, VIT_13s0019g00330, VIT_14s0108g00700, VIT_15s0021g00330, VIT_16s0050g01860, VIT_17s0000g05550*) and 3 grapevine cultivars as in (a). Linear regression analysis R^2^ value shown inset. For qRT-PCR primers see Additional file 4. Gene expression levels obtained via qRT-PCR were normalised to the geometric mean of *VvActin, VvEF1-a*, and *VvUBQ-L40*. Additional file 6:
**List of 1361 differentially expressed (** ***P*** **<0.05, ≥ ±1.41 fold) unique transcripts in at least one** ***Vitis*** **spp. genotype under 50 mM Cl** ^**−**^ **treatment.**
Additional file 7:
**Unique transcripts that were significantly up-regulated (** ***P*** **<0.05, ≥1.41 fold) in 140 Ruggeri in response to 50 mM Cl** ^**−**^ **treatment and clustering together (cluster A, Figure** 3 **).**
Additional file 8:
**Unique transcripts that were significantly down-regulated (** ***P*** **<0.05, ≤ −1.41 fold) in K51-40 in response to 50 mM Cl** ^**−**^ **treatment and clustering together (cluster B, Figure** 3 **).**
Additional file 9:
**Unique transcripts that were significantly down-regulated (** ***P*** **<0.05, ≤ −1.41 fold) in Cabernet Sauvignon in response to 50 mM Cl** ^**−**^ **treatment and clustering together (cluster C, Figure** 3 **).**
Additional file 10:
**Transcripts encoding putative membrane transporters or channels that were significantly down-regulated (** ***P*** **<0.05, ≤ −1.41 fold) in 140 Ruggeri in response to 50 mM Cl** ^**−**^ **treatment.**
Additional file 11:
**List of 4527 unique transcripts that were significantly differentially expressed (** ***P*** **<0.05, ≥ ±1.41 fold) between 140 Ruggeri and K51-40 under control conditions (non-salt).**
Additional file 12:
**List of 214 unique transcripts encoding putative membrane transporters or channels that were significantly differentially expressed (** ***P*** **<0.05, ≥ ±1.41 fold) between 140 Ruggeri and K51-40 under control conditions (non-salt).**
Additional file 13:
***VvNRT1.5*** **expression in different root tissues of grapevine.** (a) Levels of *VvNRT1.5* transcript in enriched fractions of the root cortex and stele of salt stressed (50 mM Cl^−^) grapevine determined by qRT-PCR. Bars are SEM of 3 biological replicates. Asterisk represents significant difference in the stele compared to the cortex (Student’s t-test, *P* <0.05). (b) Levels of *VvPIP1.1* transcript in enriched fractions of the root cortex and stele of salt stressed (50 mM Cl^−^) grapevine determined by qRT-PCR. Bars are SEM of 3 biological replicates. Asterisk represents significant difference in the stele compared to the cortex (Student’s t-test, *P* <0.05). *VvPIP1.1* is known to be expressed in the root stele [99], and is therefore used as a control for adequate enrichment of stele and cortex root fractions in (a).

## References

[1] Maas EV, Hoffman GJ (1977). Crop salt tolerance - current assessment. J Irrig Drain Div.

[2] Downton WJS (1977). Photosynthesis in salt-stressed grapevines. Aust J Plant Physiol.

[3] Walker R, Blackmore D, Clingeleffer P, Iacono F (1997). Effect of salinity and Ramsey rootstock on ion concentrations and carbon dioxide assimilation in leaves of drip-irrigated, field-grown grapevines (*Vitis vinifera* L. cv. Sultana). Aust J Grape Wine Res.

[4] Ehlig CF (1960). Effects of salinity on four varieties of table grapes grown in sand culture. Proceed Am Soc Horticultural Sci.

[5] Walker RR, Blackmore DH, Clingeleffer PR, Correll RL (2002). Rootstock effects on salt tolerance of irrigated field-grown grapevines (*Vitis vinifera* L. cv. Sultana). 1. Yield and vigour inter-relationships. Aust J Grape Wine Res.

[6] Zhang XK, Walker RR, Stevens RM, Prior LD (2002). Yield-salinity relationships of different grapevine (*Vitis vinifera* L.) scion-rootstock combinations. Aust J Grape Wine Res.

[7] Walker RR, Blackmore DH, Clingeleffer PR (2010). Impact of rootstock on yield and ion concentrations in petioles, juice and wine of Shiraz and Chardonnay in different viticultural environments with different irrigation water salinity. Aust J Grape Wine Res.

[8] Walker RR, Blackmore DH, Clingeleffer PR, Correll RL (2004). Rootstock effects on salt tolerance of irrigated field-grown grapevines (*Vitis vinifera* L. cv. Sultana) 2. Ion concentrations in leaves and juice. Aust J Grape Wine Res.

[9] Teakle NL, Tyerman SD (2010). Mechanisms of Cl^−^ transport contributing to salt tolerance. Plant Cell Environ.

[10] Apse MP, Aharon GS, Snedden WA, Blumwald E (1999). Salt tolerance conferred by overexpression of a vacuolar Na^+^/H^+^ antiport in *Arabidopsis*. Science.

[11] Munns R, James RA, Xu B, Athman A, Conn SJ, Jordans C, Byrt CS, Hare RA, Tyerman SD, Tester M, Plett D, Gilliham M (2012). Wheat grain yield on saline soils is improved by an ancestral Na^+^ transporter gene. Nat Biotechnol.

[12] Munns R, Tester M (2008). Mechanisms of salinity tolerance. Annu Rev Plant Physiol Plant Mol Biol.

[13] Shi H, Lee BH, Wu SJ, Zhu JK (2003). Overexpression of a plasma membrane Na^+^/H^+^ antiporter gene improves salt tolerance in *Arabidopsis thaliana*. Nat Biotechnol.

[14] Fort KP, Lowe KM, Thomas WA, Walker MA (2013). Cultural conditions and propagule type influence relative chloride exclusion in grapevine rootstocks. Am J Enol Vitic.

[15] Gong H, Blackmore D, Clingeleffer P, Sykes S, Jha D, Tester M, Walker R (2011). Contrast in chloride exclusion between two grapevine genotypes and its variation in their hybrid progeny. J Exp Bot.

[16] Tregeagle JM, Tisdall JM, Tester M, Walker RR (2010). Cl^−^ uptake, transport and accumulation in grapevine rootstocks of differing capacity for Cl^−^ exclusion. Funct Plant Biol.

[17] Köhler B, Raschke K (2000). The delivery of salts to the xylem: three types of anion conductance in the plasmalemma of the xylem parenchyma of roots of barley. Plant Physiol.

[18] Cram WJ, Pitman MG (1972). Action of abscisic acid on ion uptake and water flow in plant roots. Aust J Biol Sci.

[19] Gilliham M, Tester M (2005). The regulation of anion loading to the maize root xylem. Plant Physiol.

[20] Jia W, Wang Y, Zhang S, Zhang J (2002). Salt‐stress‐induced ABA accumulation is more sensitively triggered in roots than in shoots. J Exp Bot.

[21] Brumos J, Colmenero-Flores JM, Conesa A, Izquierdo P, Sanchez G, Iglesias DJ, Lopez-Climent MF, Gomez-Cadenas A, Talon M (2009). Membrane transporters and carbon metabolism implicated in chloride homeostasis differentiate salt stress responses in tolerant and sensitive *Citrus* rootstocks. Function Integrative Genom.

[22] Brumos J, Talon M, Bouhlal RYM, Colmenero-Flores JM (2010). Cl^−^ homeostasis in includer and excluder *Citrus* rootstocks: transport mechanisms and identification of candidate genes. Plant Cell Environ.

[23] Negi J, Matsuda O, Nagasawa T, Oba Y, Takahashi H, Kawai-Yamada M, Uchimiya H, Hashimoto M, Iba K (2008). CO2 regulator SLAC1 and its homologues are essential for anion homeostasis in plant cells. Nature.

[24] Vahisalu T, Kollist H, Wang YF, Nishimura N, Chan WY, Valerio G, Lamminmaki A, Brosche M, Moldau H, Desikan R, Schroeder JI, Kangasjarvi J (2008). SLAC1 is required for plant guard cell S-type anion channel function in stomatal signalling. Nature.

[25] Colmenero-Flores JM, Martinez G, Gamba G, Vazquez N, Iglesias DJ, Brumos J, Talon M (2007). Identification and functional characterization of cation-chloride cotransporters in plants. Plant J.

[26] Krapivinsky GB, Ackerman MJ, Gordon EA, Krapivinsky LD, Clapham DE (1994). Molecular characterization of a swelling-induced chloride conductance regulatory protein, plCIn. Cell.

[27] Jaillon O, Aury JM, Noel B, Policriti A, Clepet C, Casagrande A, Choisne N, Aubourg S, Vitulo N, Jubin C, Vezzi A, Legeai F, Hugueney P, Dasilva C, Horner D, Mica E, Jublot D, Poulain J, Bruyere C, Billault A, Segurens B, Gouyvenoux M, Ugarte E, Cattonaro F, Anthouard V, Vico V, Del Fabbro C, Alaux M, Di Gaspero G, Dumas V (2007). The grapevine genome sequence suggests ancestral hexaploidization in major angiosperm phyla. Nature.

[28] Velasco R, Zharkikh A, Troggio M, Cartwright DA, Cestaro A, Pruss D, Pindo M, FitzGerald LM, Vezzulli S, Reid J, Malacarne G, Iliev D, Coppola G, Wardell B, Micheletti D, Macalma T, Facci M, Mitchell JT, Perazzolli M, Eldredge G, Gatto P, Oyzerski R, Moretto M, Gutin N, Stefanini M, Chen Y, Segala C, Davenport C, Dematte L, Mraz A (2007). A high quality draft consensus sequence of the genome of a heterozygous grapevine variety. Plos One.

[29] Grimplet J, Deluc LG, Tillett RL, Wheatley MD, Schlauch KA, Cramer GR, Cushman JC (2007). Tissue-specific mRNA expression profiling in grape berry tissues. BMC Genomics.

[30] Guillaumie S, Fouquet R, Kappel C, Camps C, Terrier N, Moncomble D, Dunlevy J, Davies C, Boss P, Delrot S (2011). Transcriptional analysis of late ripening stages of grapevine berry. BMC Plant Biol.

[31] Wang L, Li S, Wang J, Cramer G, Dai Z, Duan W, Xu H, Wu B, Fan P (2012). Transcriptomic analysis of grape (*Vitis vinifera* L.) leaves during and after recovery from heat stress. BMC Plant Biol.

[32] Pontin M, Piccoli P, Francisco R, Bottini R, Martinez Zapater J, Lijavetzky D (2010). Transcriptome changes in grapevine (*Vitis vinifera* L.) cv. Malbec leaves induced by ultraviolet-B radiation. BMC Plant Biol.

[33] Fasoli M, Dal Santo S, Zenoni S, Tornielli GB, Farina L, Zamboni A, Porceddu A, Venturini L, Bicego M, Murino V, Ferrarini A, Delledonne M, Pezzotti M (2012). The grapevine expression atlas reveals a deep transcriptome shift driving the entire plant into a maturation program. Plant Cell Online.

[34] Tillett R, Ergul A, Albion R, Schlauch K, Cramer G, Cushman J (2011). Identification of tissue-specific, abiotic stress-responsive gene expression patterns in wine grape (*Vitis vinifera* L.) based on curation and mining of large-scale EST data sets. BMC Plant Biol.

[35] Cramer GR, Ergul A, Grimplet J, Tillett RL, Tattersall EAR, Bohlman MC, Vincent D, Sonderegger J, Evans J, Osborne C, Quilici D, Schlauch KA, Schooley DA, Cushman JC (2007). Water and salinity stress in grapevines: early and late changes in transcript and metabolite profiles. Function Integrative Genom.

[36] Tattersall EAR, Grimplet J, Deluc L, Wheatley MD, Vincent D, Osborne C, Ergul A, Lomen E, Blank RR, Schlauch KA, Cushman JC, Cramer GR (2007). Transcript abundance profiles reveal larger and more complex responses of grapevine to chilling compared to osmotic and salinity stress. Function Integrative Genom.

[37] Schachtman DP, Thomas MR (2003). A rapid method for generating sufficient amounts of uniform genotype-specific material from the woody perennial grapevine for ion transport studies. Plant Soil.

[38] Hardiman G (2004). Microarray platforms – comparisons and contrasts. Pharmacogenomics.

[39] Grimplet J, Van Hemert J, Carbonell-Bejerano P, Diaz-Riquelme J, Dickerson J, Fennell A, Pezzotti M, Martinez-Zapater J (2012). Comparative analysis of grapevine whole-genome gene predictions, functional annotation, categorization and integration of the predicted gene sequences. BMC Res Notes.

[40] Johnson WE, Li C, Rabinovic A (2007). Adjusting batch effects in microarray expression data using empirical Bayes methods. Biostatistics.

[41] Kauffmann A, Gentleman R, Huber W (2009). arrayQualityMetrics—a bioconductor package for quality assessment of microarray data. Bioinformatics.

[42] Smyth GK: **Linear models and empirical bayes methods for assessing differential expression in microarray experiments.** *Stat Appl Genet Mol Biol* 2004, **3:**ᅟ. Article3. 10.2202/1544-6115.1027 16646809

[43] Benjamini Y, Hochberg Y (1995). Controlling the false discovery rate: a practical and powerful approach to multiple testing. J R Stat Soc Ser B Methodol.

[44] Sturn A, Quackenbush J, Trajanoski Z (2002). Genesis: cluster analysis of microarray data. Bioinformatics.

[45] Du Z, Zhou X, Ling Y, Zhang Z, Su Z (2010). agriGO: a GO analysis toolkit for the agricultural community. Nucleic Acids Res.

[46] Larkin MA, Blackshields G, Brown NP, Chenna R, McGettigan PA, McWilliam H, Valentin F, Wallace IM, Wilm A, Lopez R, Thompson JD, Gibson TJ, Higgins DG (2007). Clustal W and Clustal X version 2.0. Bioinformatics.

[47] Pfaffl MW (2001). A new mathematical model for relative quantification in real-time RT-PCR. Nucleic Acids Res.

[48] Vandesompele J, De Preter K, Pattyn F, Poppe B, Van Roy N, De Paepe A, Speleman F (2002). Accurate normalization of real-time quantitative RT-PCR data by geometric averaging of multiple internal control genes. Genome Biol.

[49] Untergasser A, Cutcutache I, Koressaar T, Ye J, Faircloth BC, Remm M, Rozen SG (2012). Primer3 - new capabilities and interfaces. Nucleic Acids Res.

[50] Roxas VP, Smith RK, Allen ER, Allen RD (1997). Overexpression of glutathione S-transferase/glutathioneperoxidase enhances the growth of transgenic tobacco seedlings during stress. Nat Biotechnol.

[51] Sun W, Van Montagu M, Verbruggen N (2002). Small heat shock proteins and stress tolerance in plants. Biochim Biophys Acta (BBA) Gene Struct Express.

[52] Hassan S, Mathesius U (2012). The role of flavonoids in root–rhizosphere signalling: opportunities and challenges for improving plant–microbe interactions. J Exp Bot.

[53] Abogadallah GM (2010). Insights into the significance of antioxidative defense under salt stress. Plant Signal Behav.

[54] Barbier-Brygoo H, De Angeli A, Filleur S, Frachisse J-M, Gambale F, Thomine S, Wege S (2011). Anion channels/transporters in plants: from molecular bases to regulatory networks. Annu Rev Plant Biol.

[55] Tsay YF, Chiu CC, Tsai CB, Ho CH, Hsu PK (2007). Nitrate transporters and peptide transporters. Febs Lett.

[56] Li JY, Fu YL, Pike SM, Bao J, Tian W, Zhang Y, Chen CZ, Zhang Y, Li HM, Huang J, Li LG, Schroeder JI, Gassmann W, Gong JM (2010). The *Arabidopsis* nitrate transporter NRT1.8 functions in nitrate removal from the xylem sap and mediates cadmium tolerance. Plant Cell.

[57] Lin SH, Kuo HF, Canivenc G, Lin CS, Lepetit M, Hsu PK, Tillard P, Lin HL, Wang YY, Tsai CB, Gojon A, Tsay YF (2008). Mutation of the *Arabidopsis* NRT1.5 nitrate transporter causes defective root-to-shoot nitrate transport. Plant Cell.

[58] Chen CZ LVXF, Li JY, Yi HY, Gong JM (2012). Arabidopsis NRT1.5 is another essential component in the regulation of nitrate reallocation and stress tolerance. Plant Physiol.

[59] Segonzac C, Boyer JC, Ipotesi E, Szponarski W, Tillard P, Touraine B, Sommerer N, Rossignol M, Gibrat R (2007). Nitrate efflux at the root plasma membrane: identification of an *Arabidopsis* excretion transporter. Plant Cell.

[60] Sasaki T, Yamamoto Y, Ezaki B, Katsuhara M, Ahn SJ, Ryan PR, Delhaize E, Matsumoto H (2004). A wheat gene encoding an aluminum-activated malate transporter. Plant J.

[61] Piñeros MA, Cançado GMA, Maron LG, Lyi SM, Menossi M, Kochian LV (2008). Not all ALMT1-type transporters mediate aluminum-activated organic acid responses: the case of ZmALMT1 – an anion-selective transporter. Plant J.

[62] De Angeli A, Zhang J, Meyer S, Martinoia E (2013). AtALMT9 is a malate-activated vacuolar chloride channel required for stomatal opening in *Arabidopsis*. Nat Commun.

[63] Conn SJ, Gilliham M, Athman A, Schreiber AW, Baumann U, Moller I, Cheng N-H, Stancombe MA, Hirschi KD, Webb AAR, Burton R, Kaiser BN, Tyerman SD, Leigh RA (2011). Cell-specific vacuolar calcium storage mediated by CAX1 regulates apoplastic calcium concentration, gas exchange, and plant productivity in *Arabidopsis*. Plant Cell Online.

[64] Zhao J, Barkla BJ, Marshall J, Pittman JK, Hirschi KD (2008). The *Arabidopsis cax3* mutants display altered salt tolerance, pH sensitivity and reduced plasma membrane H^+^-ATPase activity. Planta.

[65] Geisler M, Frangne N, Gomès E, Martinoia E, Palmgren MG (2000). The *ACA4* gene of Arabidopsis encodes a vacuolar membrane calcium pump that improves salt tolerance in yeast. Plant Physiol.

[66] Brandt B, Brodsky DE, Xue S, Negi J, Iba K, Kangasjärvi J, Ghassemian M, Stephan AB, Hu H, Schroeder JI (2012). Reconstitution of abscisic acid activation of SLAC1 anion channel by CPK6 and OST1 kinases and branched ABI1 PP2C phosphatase action. Proc Natl Acad Sci.

[67] Hedrich R (2012). Ion channels in plants. Physiol Rev.

[68] Song W-Y, Park J, Mendoza-Cózatl DG, Suter-Grotemeyer M, Shim D, Hörtensteiner S, Geisler M, Weder B, Rea PA, Rentsch D, Schroeder JI, Lee Y, Martinoia E (2010). Arsenic tolerance in *Arabidopsis* is mediated by two ABCC-type phytochelatin transporters. Proc Natl Acad Sci.

[69] Lee EK, Kwon M, Ko J-H, Yi H, Hwang MG, Chang S, Cho MH (2004). Binding of sulfonylurea by AtMRP5, an *Arabidopsis* multidrug resistance-related protein that functions in salt tolerance. Plant Physiol.

[70] Cotsaftis O, Plett D, Johnson AAT, Walia H, Wilson C, Ismail AM, Close TJ, Tester M, Baumann U (2011). Root-specific transcript profiling of contrasting rice genotypes in response to salinity stress. Mol Plant.

[71] Viehweger K, Dordschbal B, Roos W (2002). Elicitor-activated phospholipase A2 generates lysophosphatidylcholines that mobilize the vacuolar H^+^ pool for pH signaling via the activation of Na^+^-dependent proton fluxes. Plant Cell Online.

[72] Cramer G, Urano K, Delrot S, Pezzotti M, Shinozaki K (2011). Effects of abiotic stress on plants: a systems biology perspective. BMC Plant Biol.

[73] Mittler R, Vanderauwera S, Suzuki N, Miller G, Tognetti VB, Vandepoele K, Gollery M, Shulaev V, Van Breusegem F (2011). ROS signaling: the new wave?. Trends Plant Sci.

[74] Ismail A, Riemann M, Nick P (2012). The jasmonate pathway mediates salt tolerance in grapevines. J Exp Bot.

[75] Walia H, Wilson C, Condamine P, Ismail A, Xu J, Cui X, Close T (2007). Array-based genotyping and expression analysis of barley cv. *Maythorpe Golden Promise*. BMC Genom.

[76] Walia H, Wilson C, Wahid A, Condamine P, Cui X, Close T (2006). Expression analysis of barley (*Hordeum vulgare* L.) during salinity stress. Funct Integrative Genom.

[77] Wang Y, Yang L, Zheng Z, Grumet R, Loescher W, Zhu J-K, Yang P, Hu Y, Chan Z (2013). Transcriptomic and physiological variations of three *Arabidopsis* ecotypes in response to salt stress. PLoS ONE.

[78] Geiger D, Maierhofer T, AL-Rasheid KAS, Scherzer S, Mumm P, Liese A, Ache P, Wellmann C, Marten I, Grill E, Romeis T, Hedrich R (2011). Stomatal closure by fast abscisic acid signaling is mediated by the guard cell anion channel SLAH3 and the receptor RCAR1. Sci Signal.

[79] Meyer S, Mumm P, Imes D, Endler A, Weder B, Al-Rasheid KAS, Geiger D, Marten I, Martinoia E, Hedrich R (2010). AtALMT12 represents an R-type anion channel required for stomatal movement in *Arabidopsis* guard cells. Plant J.

[80] White PJ, Broadley MR (2001). Chloride in soils and its uptake and movement within the plant: a review. Ann Bot.

[81] Imes D, Mumm P, Böhm J, Al-Rasheid KAS, Marten I, Geiger D, Hedrich R (2013). Open stomata 1 (OST1) kinase controls R–type anion channel QUAC1 in *Arabidopsis* guard cells. Plant J.

[82] Gutermuth T, Lassig R, Portes M-T, Maierhofer T, Romeis T, Borst J-W, Hedrich R, Feijó JA, Konrad KR (2013). Pollen tube growth regulation by free anions depends on the interaction between the anion channel SLAH3 and calcium-dependent protein kinases CPK2 and CPK20. Plant Cell Online.

[83] Sykes SR (1985). Variation in chloride accumulation by hybrid vines from crosses involving the cultivars Ramsey, Villard Blanc, and Sultana. Am J Enol Vitic.

[84] Deane-Drummond CE (1986). A comparison of regulatory effects of chloride on nitrate uptake, and of nitrate on chloride uptake into *Pisum sativum* seedlings. Physiol Plant.

[85] Glass ADM, Siddiqi MY (1985). Nitrate inhibition of chloride influx in barley - implications for a proposed chloride homeostat. J Exp Bot.

[86] Jossier M, Kroniewicz L, Dalmas F, Le Thiec D, Ephritikhine G, Thomine S, Barbier-Brygoo H, Vavasseur A, Filleur S, Leonhardt N (2010). The *Arabidopsis* vacuolar anion transporter, AtCLCc, is involved in the regulation of stomatal movements and contributes to salt tolerance. Plant J.

[87] Li W-YF, Wong F-L, Tsai S-N, Phang T-H, Shao G, Lam H-M (2006). Tonoplast-located GmCLC1 and GmNHX1 from soybean enhance NaCl tolerance in transgenic bright yellow (BY)-2 cells. Plant Cell Environ.

[88] Nakamura A, Fukuda A, Sakai S, Tanaka Y (2006). Molecular cloning, functional expression and subcellular localization of two putative vacuolar voltage-gated chloride channels in rice (*Oryza sativa* L.). Plant Cell Physiol.

[89] Wei Q, Liu Y, Zhou G, Li Q, Yang C, Peng SA (2013). Overexpression of *CsCLCc,* a chloride channel gene from *Poncirus trifoliata*, enhances salt tolerance in *Arabidopsis*. Plant Mol Biol Report.

[90] Von der Fecht-Bartenbach J, Bogner M, Dynowski M, Ludewig U (2010). CLC-b-mediated NO_3_^−^/H^+^ exchange across the tonoplast of *Arabidopsis* vacuoles. Plant Cell Physiol.

[91] De Angeli A, Monachello D, Ephritikhine G, Frachisse JM, Thomine S, Gambale F, Barbier-Brygoo H (2006). The nitrate/proton antiporter AtCLCa mediates nitrate accumulation in plant vacuoles. Nature.

[92] Bergsdorf E-Y, Zdebik AA, Jentsch TJ (2009). Residues important for nitrate/proton coupling in plant and mammalian CLC transporters. J Biol Chem.

[93] Wege S, Jossier M, Filleur S, Thomine S, Barbier-Brygoo H, Gambale F, De Angeli A (2010). The proline 160 in the selectivity filter of the *Arabidopsis* NO_3_^−^/H^+^ exchanger AtCLCa is essential for nitrate accumulation in planta. Plant J.

[94] Marmagne A, Vinauger-Douard M, Monachello D, de Longevialle AF, Charon C, Allot M, Rappaport F, Wollman F-A, Barbier-Brygoo H, Ephritikhine G (2007). Two members of the *Arabidopsis* CLC (chloride channel) family, AtCLCe and AtCLCf, are associated with thylakoid and Golgi membranes, respectively. J Exp Bot.

[95] Liu KH, Tsay YF (2003). Switching between the two action modes of the dual‐affinity nitrate transporter CHL1 by phosphorylation. J Article.

[96] Lee SC, Lan W, Buchanan BB, Luan S (2009). A protein kinase-phosphatase pair interacts with an ion channel to regulate ABA signaling in plant guard cells. Proc Natl Acad Sci.

[97] Wege S, De Angeli A, Droillard M-J, Kroniewicz L, Merlot S, Cornu D, Gambale F, Martinoia E, Barbier-Brygoo H, Thomine S, Leonhardt N, Filleur S (2014). Phosphorylation of the vacuolar anion exchanger AtCLCa is required for the stomatal response to abscisic acid. Sci Signal.

[98] Tregeagle JM, Tisdall JM, Blackmore DH, Walker RR (2006). A diminished capacity for chloride exclusion by grapevine rootstocks following long-term saline irrigation in an inland versus a coastal region of Australia. Aust J Grape Wine Res.

[99] Vandeleur RK, Mayo G, Shelden MC, Gilliham M, Kaiser BN, Tyerman SD (2009). The role of plasma membrane intrinsic protein aquaporins in water transport through roots: diurnal and drought stress responses reveal different strategies between isohydric and anisohydric cultivars of grapevine. Plant Physiol.

