# Supplementary figures and images for: Shoot chloride exclusion and salt tolerance in grapevine is associated with differential ion transporter expression in roots
Source: BMC Plant Biol. 2014 Oct 25;14:273. doi: 10.1186/s12870-014-0273-8 (PMC4220414; doi:10.1186/s12870-014-0273-8)

**Additional file 3**


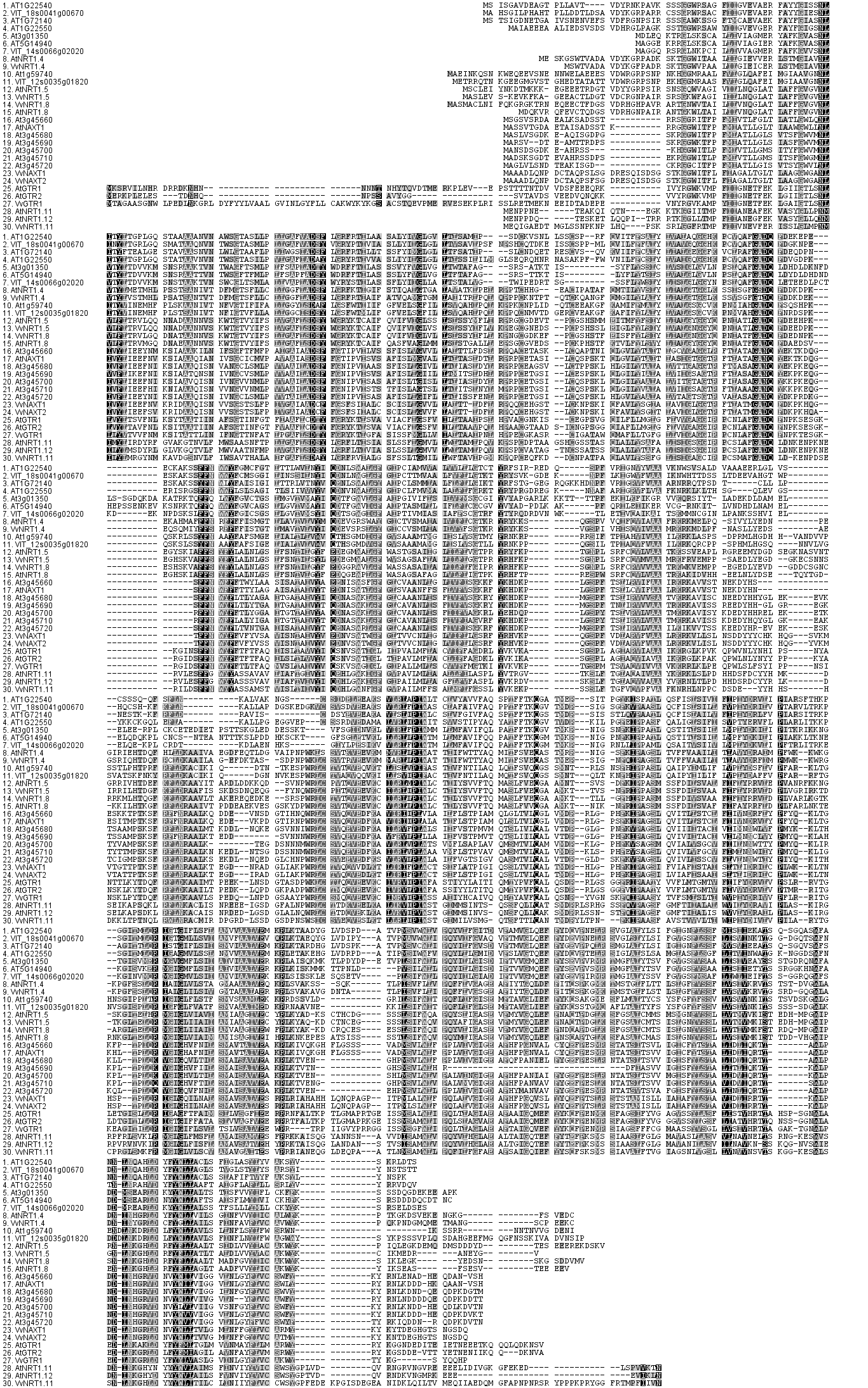

Supplement: Additional file 3: — Multiple sequence alignment of Arabidopsis and grapevine NRT members for the data presented in Figure 4 . Shading is representative of the Blosum62 score matrix as follows: black (100% similar) dark grey (80% similar) light grey (60% similar) unshaded (<60% similar). [file 12870_2014_273_MOESM3_ESM.docx]

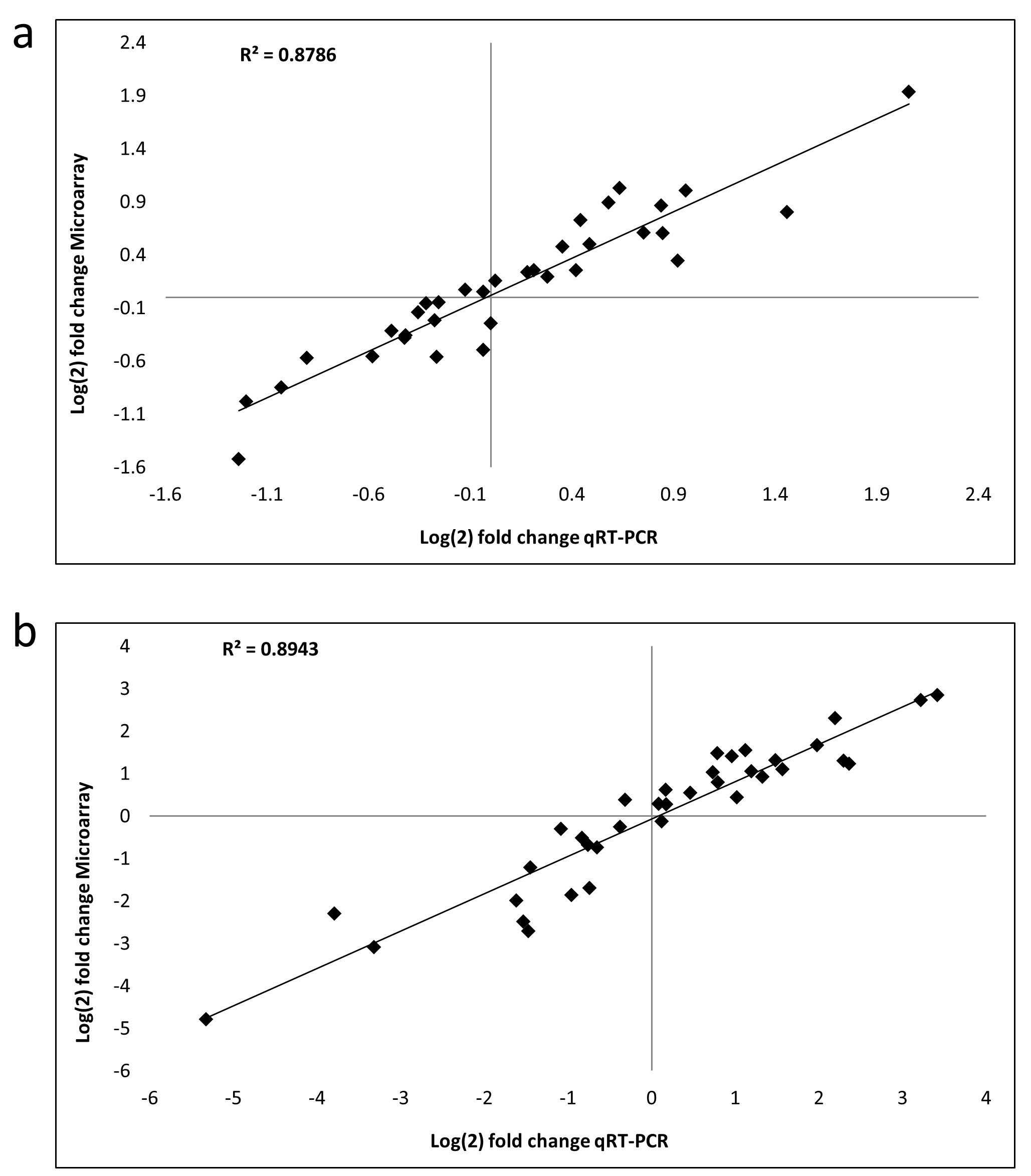

Supplement: Additional file 5: — Correlation between expression ratios determined by Agilent custom gene expression array and qRT-PCR. (a) Gene expression ratios of control to salt treated samples were compared for 12 genes (VIT_00s0229g00130, VIT_01s0011g06550, VIT_02s0012g01160, VIT_06s0004g03520, VIT_06s0080g00170, VIT_08s0040g01890, VIT_08s0040g03220, VIT_11s0016g05170, VIT_13s0019g00330, VIT_14s0108g00700, VIT_15s0021g00330, VIT_16s0050g01860) in all 3 grapevine varieties by microarray and qRT-PCR. (b) Gene expression ratios of varietal differences under control conditions were compared for 12 genes (VIT_01s0011g06550, VIT_02s0012g01160, VIT_06s0004g03520, VIT_06s0080g00170, VIT_08s0040g01890, VIT_08s0040g03220, VIT_11s0016g05170, VIT_13s0019g00330, VIT_14s0108g00700, VIT_15s0021g00330, VIT_16s0050g01860, VIT_17s0000g05550) and 3 grapevine cultivars as in (a). Linear regression analysis R2 value shown inset. For qRT-PCR primers see Additional file 4. Gene expression levels obtained via qRT-PCR were normalised to the geometric mean of VvActin, VvEF1-a, and VvUBQ-L40. [file 12870_2014_273_MOESM5_ESM.tiff]
